# Predicting *Wolbachia* invasion dynamics in *Aedes aegypti* populations using models of density-dependent demographic traits

**DOI:** 10.1186/s12915-016-0319-5

**Published:** 2016-11-08

**Authors:** Penelope A. Hancock, Vanessa L. White, Scott A. Ritchie, Ary A. Hoffmann, H. Charles J. Godfray

**Affiliations:** 1 Department of Zoology, University of Oxford, South Parks Road, Oxford, OX1 3PS UK; 2 Bio 21 Institute, School of Biosciences, University of Melbourne, Melbourne, Victoria 3010 Australia; 3 Australian Institute of Tropical Health and Medicine, James Cook University, Townsville City, Queensland 4878 Australia

**Keywords:** *Wolbachia*, Zika, Dengue, Mosquito, *Aedes aegypti*, Density-dependence, Bayesian statistical model, Invasion, Vector-borne disease, Fitness

## Abstract

**Background:**

Arbovirus transmission by the mosquito *Aedes aegypti* can be reduced by the introduction and establishment of the endosymbiotic bacteria *Wolbachia* in wild populations of the vector. *Wolbachia* spreads by increasing the fitness of its hosts relative to uninfected mosquitoes. However, mosquito fitness is also strongly affected by population size through density-dependent competition for limited food resources. We do not understand how this natural variation in fitness affects symbiont spread, which limits our ability to design successful control strategies.

**Results:**

We develop a mathematical model to predict *A. aegypti–Wolbachia* dynamics that incorporates larval density-dependent variation in important fitness components of infected and uninfected mosquitoes. Our model explains detailed features of the mosquito–*Wolbachia* dynamics observed in two independent experimental *A. aegypti* populations, allowing the combined effects on dynamics of multiple density-dependent fitness components to be characterized. We apply our model to investigate *Wolbachia* field release dynamics, and show how invasion outcomes can depend strongly on the severity of density-dependent competition at the release site. Specifically, the ratio of released relative to wild mosquitoes required to attain a target infection frequency (at the end of a release program) can vary by nearly an order of magnitude. The time taken for *Wolbachia* to become established following releases can differ by over 2 years. These effects depend on the relative fitness of field and insectary-reared mosquitoes.

**Conclusions:**

Models of *Wolbachia* invasion incorporating density-dependent demographic variation in the host population explain observed dynamics in experimental *A. aegypti* populations. These models predict strong effects of density-dependence on *Wolbachia* dynamics in field populations, and can assist in the effective use of *Wolbachia* to control the transmission of arboviruses such as dengue, chikungunya and zika.

**Electronic supplementary material:**

The online version of this article (doi:10.1186/s12915-016-0319-5) contains supplementary material, which is available to authorized users.

## Background

The introduction of self-spreading infections of the endosymbiotic bacteria *Wolbachia* into field populations of *Aedes aegypti* mosquitoes is a novel strategy for the biocontrol of arboviruses that is currently undergoing field trials in disease-affected regions across multiple countries [1]. Infection with *Wolbachia* reduces the capacity of *A. aegypti* to transmit dengue [2, 3] and other arboviruses, including zika [4] and chikungunya [5]. Field releases of mosquitoes infected with the *Wolbachia* strain *w*Mel have achieved stable establishment of the bacteria in wild *A. aegypti* populations in north-east Australia [6]. Further field trials aim to establish *w*Mel in areas of south-east Asia and South America within geographic regions that bear the highest dengue burden [7]. In applying the strategy in these new environmental settings, field releases will need to scale up to larger urban areas. Large urban settlements with high human and mosquito densities are important in driving dengue transmission [8], and present more challenging conditions for *Wolbachia* invasion compared to the low-density populations in north-east Australia.

The time taken for *Wolbachia* infections to become established has varied across recent field release trials, and establishment has failed to occur in some cases. *w*Mel establishment occurred within 2 months following releases in north-east Australia [6], while invasion in Indonesia has been slower, with frequencies remaining below 90 % for several months following releases, despite over 60 % of mosquitoes in the field being infected during the release period (Warsito Tantowijoyo *pers. comm.*). In Brazil, *w*Mel frequencies have declined to low levels following releases, again despite the attainment of a high percentage infection (65 %) during the release period [9].

In order to design release strategies that achieve successful *Wolbachia* invasion across a range of environmental settings, it is critical to develop an understanding of the factors influencing *Wolbachia* invasion in wild mosquito populations. *Wolbachia* is maternally transmitted and it invades by manipulating host reproduction, most commonly using a mechanism known as cytoplasmic incompatibility. Cytoplasmic incompatibility in *A. aegypti* transfected by *w*Mel causes near-complete non-viability of offspring from matings between uninfected females and infected males [3], conferring a relative fitness advantage to infected females [10]. This advantage is stronger when the *Wolbachia* frequency in the insect population is higher, and simple population genetic models predict a threshold frequency below which invasion does not occur [11].

Most studies of *Wolbachia* invasion have assumed that host demographic rates, and the relative fitness of infected individuals, are constant and independent of population density (e.g., [3, 6, 11] but see [12, 13]). However, in *A. aegypti*, experimental studies have shown that fecundity, and juvenile survival and development rates, vary strongly depending on the level of larval density-dependent competition for food [14–17]. Larval density-dependent demographic variation in field populations has proven difficult to quantify because mosquito populations have overlapping generations [15, 16, 18]. However, field observations of *A. aegypti* suggest that competition is often relatively intense. The body size of adults emerging from field-collected pupae is typically significantly smaller than that of individuals experiencing plentiful food in the laboratory [12, 19, 20], and similar to those developing under conditions of strong resource competition [21] where fecundity is reduced [22]. Variation in these important fitness components may strongly affect *Wolbachia* spread [17]. It is thus important to develop our understanding of density-dependent fitness in *A. aegypti*, as well as any differential effects on infected individuals, in order to increase our capacity to predict and facilitate *Wolbachia* invasion.

*Wolbachia*-infected mosquitoes that are reared in an insectary and then released into the field may be disadvantaged if they lack adaptations to the local environment. For example, the released infected adults may experience higher susceptibility to chemical insecticides. Although *Wolbachia* is not expected to directly influence insecticide resistance, released mosquitoes may be more susceptible because of the genetic background of *Wolbachia-*infected insectary *A. aegypti* colonies [23]. Backcrossing of these colonies with wild-collected mosquitoes can counteract this disadvantage [6], but these procedures may be inadequate, particularly if insecticide resistance incurs a fitness cost in the absence of insecticides [24]. There are also ethical issues concerning the release of insecticide-resistant vectors. Reduced insecticide resistance in infected mosquitoes has been proposed as a reason for the failure of *w*Mel establishment following field releases in Brazil [9] because of the local heavy application of chemical insecticides [25]. We need an improved understanding of natural mosquito fitness variation in wild populations in order to predict the impacts of such fitness disadvantages on *Wolbachia* invasion.

Here, we develop a mathematical model to predict the effects of larval density-dependent demographic variation on the dynamics of both *A. aegypti* and *w*Mel *Wolbachia*. Our model incorporates mathematical relationships describing density-dependent demographic traits in infected and uninfected mosquitoes parameterized using observations from two independent field-cage experimental populations. We simulate *w*Mel dynamics following field releases, focusing on a practical target that requires releases to rapidly achieve a high infection frequency. We explore the consequences of released mosquitoes suffering different degrees of fitness disadvantage as a result of lacking relevant genetic adaptations, as would occur if they were less resistant to insecticides than the native mosquitoes. We demonstrate how density-dependent variation in demographic traits can strongly influence interactions between mosquito fitness and *Wolbachia* frequency dynamics, greatly impacting the size of release required to meet our target frequency as well as the time taken for *Wolbachia* to become established following releases. *Wolbachia* is a promising candidate to help control diseases spread by *A. aegypti*, and our models, incorporating important features of natural population dynamics, can assist in developing effective field release strategies.

## Results

**Fig. 1:**
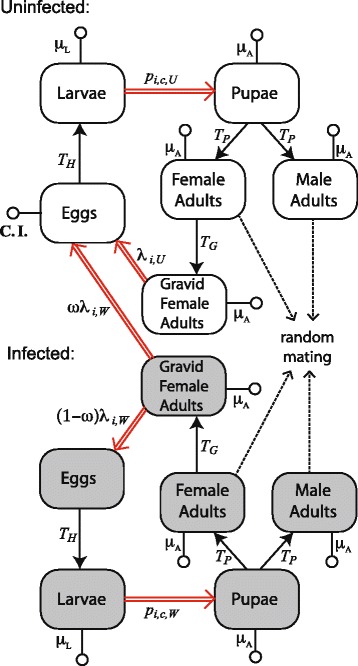
Model of mosquito–*Wolbachia* dynamics. Double red arrows indicate demographic rates that depend on larval density, solid arrows indicate fixed time lags, dotted arrows indicate mating interactions, and open circle terminators indicate mortality rates. Grey shading and no shading indicate *Wolbachia*-infected and uninfected life stages, respectively. Parameter symbols and values are defined in the text and Additional file 1: Table S1.1. The computer code for implementing our model (gyp_sim.cpp) has been made available (see doi 10.6084/m9.figshare.3980472)

Our model of mosquito–*Wolbachia* dynamics allows two mosquito demographic traits, namely per-capita female fecundity and larval development times, to vary with changing larval density (Fig. 1; double red lines). We express these demographic traits as functions of larval density and allow their values to differ between infected and uninfected mosquitoes (see Methods). We estimate the form of these functions using observations from two independent populations of *A. aegypti* housed in field-cages (see below). The other demographic traits defined in our model (Fig. 1) are assumed to be density and time independent, and are estimated from direct observations either from our work or from previous studies. Details of the models and data are provided in the Methods.

### Estimating density-dependent demographic traits

We refer to our two experimental field-cage *A. aegypti* populations as Population A (see [17]) and Population B (see Methods). *w*Mel *Wolbachia* was introduced into both populations but in different ways. Population A was initiated with a cohort of uninfected adults and then at 2 months we began regular introductions of *w*Mel-infected adults produced from larvae which had been reared in a separate facility (see Methods). Population B was initiated with a cohort of which 40 % of the adults were infected with *w*Mel, and this population then received no further introductions of infected mosquitoes. The parameters of the functions describing density-dependent demographic traits were estimated using Bayesian Markov Chain Monte Carlo (MCMC) methods informed by (1) our observed abundances of the juvenile mosquito life stages, and (2) our observed *w*Mel infection frequencies in first instar larvae and pupae over time in our two populations (see Methods).

#### Larval development times

**Fig. 2:**
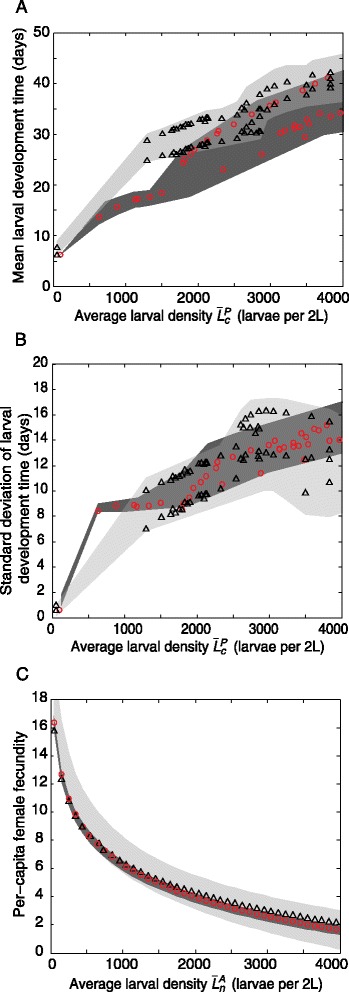
Posterior fitted values of larval density-dependent mosquito demographic traits for two field-cage populations. **a** Mean larval development time. **b** Standard deviation of larval development times. **c** Per-capita female fecundity. Red circles and black triangles show the values corresponding to the maximum posterior probability for Populations A and B, respectively. Dark and light grey shaded areas show the 95 % credible interval for Populations A and B, respectively, and mid-grey shaded areas show regions where the credible intervals for the two populations overlap. The measures of average larval density, ${\overline{L}}_c^P$ and ${\overline{L}}_n^A$, are defined in the Methods

The mean development times of the larval cohorts are much longer at higher larval densities, with similar fitted models describing this relationship for both populations (Fig. 2a). The variation in development times is also greater at higher larval densities, with both populations again showing similar relationships (Fig. 2b). The fitted models of density-dependent larval development times explain the major features of the dynamics of mosquito numbers and *Wolbachia* infection frequencies as measured by daily pupal surveys (Additional file 3: Figure S1.1, Additional file 4: Figure S1.2 and Additional file 2: Text S1).

**Fig. 3:**
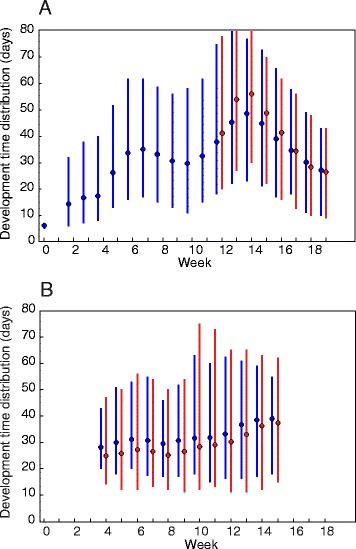
Predicted development time distributions of infected and uninfected larvae. **a** Population A; **b** Population B. Circles show the mean and vertical lines connect the 5 % and 95 % quantiles for uninfected (blue) and infected (red) larvae hatched in each week. The MCMC iteration with the maximum posterior probability is shown

For both populations, the predicted larval development times are highly variable amongst the individuals hatched in each cohort (Fig. 3). For Population A, the predicted mean development times of infected and uninfected larvae did not differ significantly (95 % credible interval (CI) includes 0; see Methods and Additional file 6: Figure S2.1). However, for Population B infected larvae developed faster than uninfected larvae for cohorts hatched in the first 5 weeks (Additional file 6: Figure S2.1; 95 % CI > 0 for cohorts hatched in weeks 4–8). For subsequent cohorts (hatched in weeks 9–15), the predicted mean development times of infected and uninfected larvae did not differ significantly. The faster development of infected larvae in the earlier cohorts may possibly be caused by genetic differences between the infected and uninfected mosquitoes (see the Discussion).

#### Per-capita female fecundity

Per-capita female fecundity declined strongly (by about factor of 10) with increasing larval density, with similar fitted models describing this relationship for both populations (Fig. 2c). Models incorporating these density-dependent relationships describe the major features of the dynamics of the average number of hatched larvae per week for both populations (Additional file 5: Figure S1.3), except for a short period in Population B (weeks 11–15), which is explored in Additional file 2: Text S1. For Population B, the predicted per-capita female fecundity over time did not differ significantly between infected and uninfected adults (95 % CI includes 0; Additional file 8: Figure S3.2, Additional file 7: Figure S3.1). For Population A, our data allow estimation of the per-capita fecundity over time of uninfected, but not infected, adult females (see Methods and [17]).

### Modelling density-dependent population dynamics

We incorporated these estimates of density-dependent mosquito demographic traits into our model of mosquito–*Wolbachia* dynamics (Fig. 1 and Methods). We first use the model to predict their values when the mosquito population is at equilibrium and *Wolbachia* is not present. At equilibrium, the per-capita female fecundity *λ* ^*^ and the larval development time distribution ${\tilde{T}}_L^{*}$ depend on the level of larval density-dependent competition. We define the equilibrium net larval survival (from first instar to pupal eclosion) to be ${\theta}_L\left({\tilde{T}}_L^{*},{\mu}_L\right)$, which depends on the mortality experienced throughout the larval stage, which we assume here occurs at a constant daily rate, *μ*_*L*_. Then, at equilibrium,

1 $$ \frac{\lambda^{*}{\theta}_L\left({\tilde{T}}_L^{*},{\mu}_L\right){\theta}_P{\theta}_{A_G}}{\mu_A}=1 $$

where *θ*_*P*_ and ${\theta}_{A_G}$ are the probabilities of surviving through the pupal stage and the early adult stage (during which females are too young to produce eggs), respectively (see Additional file 2: Text S1 and [26]). This expression simply states that each adult female produces, on average, one adult female offspring throughout its lifetime. Therefore, when the population experiences higher juvenile or adult mortality (higher *μ*_*L*_ or *μ*_*A*_), the intensity of density-dependent competition at equilibrium decreases through changes in *λ* ^*^and ${\tilde{T}}_L^{*}$.

Field mosquito populations are expected to experience higher density-independent mortality than our experimental field-cage populations, though mortality rates of juveniles and adults in the field are uncertain [6, 18]. We assume that the field population experiences density-independent mortality at a constant daily rate, *μ*_*I*_, during both the larval and adult stages. We further assume that this mortality acts in addition to the mortality occurring in our experimental populations, so that *μ*_*A*_ = *μ*_*A*_^*c*^ + *μ*_*I*_ and *μ*_*L*_ = *μ*_*L*_^*c*^ + *μ*_*I*_, where *μ*_*A*_^*c*^ and *μ*_*L*_^*c*^ are the daily juvenile and adult mortality rates in our field-cage populations, respectively (Fig. 1 and Additional file 1: Table S1.1)*.* We define the intensity of density-dependent competition experienced in the field population at equilibrium relative to the equilibrium derived from the field-cage experiments as *C*_I_^*^ = *L*_I_^*^/*L*_0_^*^ where *L*_I_^*^ and *L*_0_^*^ are the equilibrium larval densities given by a fixed value of *μ*_*I*_ ≥ 0 and *μ*_*I*_ = 0, respectively.

**Fig. 4:**
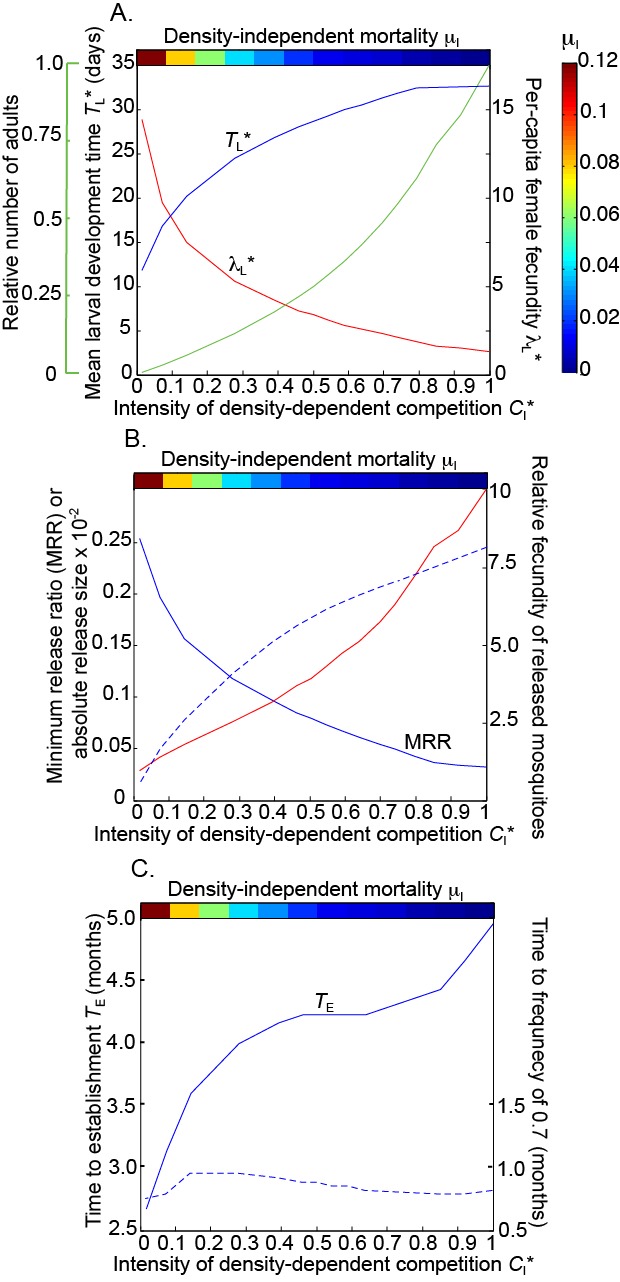
Mosquito demographic traits and *Wolbachia* field release dynamics depend strongly on the intensity of density dependence. **a** Equilibrium values of the mean larval development time (blue line), the per-capita female fecundity (red line) and the number of adults relative to the value when *C*_I_^*^ = 1 (green line). **b** The fecundity of released relative to wild mosquitos (red line), the minimum release ratio (solid blue line) and the absolute number of released mosquitoes (dotted blue line). **c** The time to *Wolbachia* establishment (*T*_*E*_; solid line) for different intensities of density-dependent competition *C*_I_^*^. The dotted line shows the time taken for the *Wolbachia* frequency to reach 0.7 following the final release

As we increase the level of density-independent mortality, *μ*_*I*_, the intensity of larval competition declines and the equilibrium values of both the per-capita female fecundity (Fig. 4a; red line) and the mean larval development time (Fig. 4a; blue line) vary across the range of values observed in our experimental populations. Higher mortality, *μ*_*I*_, has a stronger effect on population size than the increased density-dependent fitness, and causes a steep decline in equilibrium population size (Fig. 4a; green line).

### Predicting *Wolbachia* dynamics following field releases

We now explore how differences in the intensity of density-dependent competition in the field population affect the dynamics of *Wolbachia* following release. We model a typical strategy used in actual campaigns, where a fixed number of mosquitoes infected with *w*Mel is released every week over 3 months [6, 9] and assume that the field population is at equilibrium when releases commence. The absolute number of mosquitoes that need to be released to achieve a given infection frequency is determined by the “release ratio”, or the size of each release divided by the initial wild population size. We set as a target that the frequency of infected adults must exceed 0.6 one week after the final release [3, 6] and calculate the minimum release ratio (MRR) required to achieve the target frequency. We then obtain the time taken for the *Wolbachia* to become established following the final release, *T*_*E*_, given that the release ratio is equal to the MRR, and defining establishment to occur when the infection frequency exceeds 0.95.

If larvae in the field population experience significant density-dependent competition, then it is likely that the released mosquitoes, reared with plentiful food, will have higher average female fecundity. If the average fecundity of the released females is equal to that which we observed at the lowest larval density (Fig. 2c), then they will have a very strong fecundity advantage (Fig. 4b; red line), especially when the intensity of density-dependent competition in the field population (*C*_I_^*^) is high. Therefore, the MRR increases by nearly an order of magnitude across decreasing intensities of competition *C*_I_^*^ (Fig. 4b; solid blue line).

However, when competition is more intense, greater absolute numbers of released mosquitoes are required to meet the target frequency (Fig. 4b; dotted blue line) even though the MRR is lower. This is because the size of the field population is much larger due to the low density-independent mortality (Fig. 4a). Further, larval development periods are longer under more intense competition, which slows *Wolbachia* spread [17]. Therefore, the time to *Wolbachia* establishment following releases (*T*_*E*_) is longer (Fig. 4c). Thus, situations where competition is intense are disadvantageous for field release strategies overall, despite released females having a much stronger fitness advantage.

#### Fitness disadvantages in released mosquitoes

We now consider the situation where released mosquitoes are at a fitness disadvantage compared to wild-type individuals. Specifically, we assume that released individuals are more susceptible to a chemical insecticide used widely at the release site, leading to reduced adult survival. We assume that this fitness disadvantage is independent of *Wolbachia* infection so the disadvantage experienced by infected individuals declines over the generations following release due to introgression of wild-type genes. We assume that resistance is encoded by one allele at a single nuclear locus (R, resistant; S, susceptible) giving three genotypes: RR, SR, and SS. Homozygote resistant individuals are unaffected by the insecticide. Homozygote susceptible individuals experience a proportional reduction in daily adult survival of 1-*c*_*SS*_, with the cost, *c*_*SS*_ = 0.8, assumed to be substantial. Heterozygote survival is reduced by a fraction of this amount to 1-*f*_*SR*_ *c*_*SS*_ (see Methods and [27]), where 0 ≤ *f*_*SR*_ ≤ 1. The field population prior to releases is assumed to be entirely composed of homozygote resistant individuals and all released mosquitoes are assumed to have the homozygote susceptible genotype. As before, we assume that the per-capita fecundity of the released mosquitoes is high, equal to that observed at the lowest larval densities in the field-cages.

**Fig. 5:**
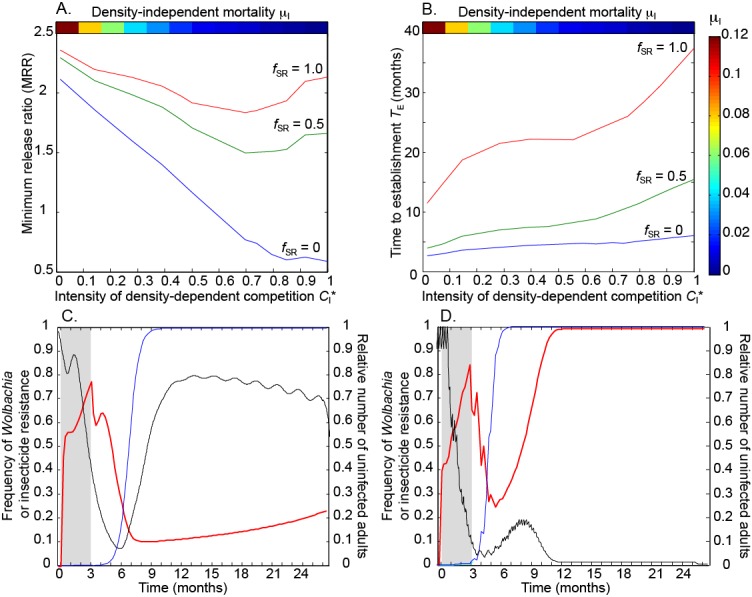
Effects of density-dependence on *Wolbachia* dynamics are enhanced when field-released mosquitoes experience fitness disadvantages. **a** The minimum release ratio. **b** The time to *Wolbachia* establishment. Released mosquitoes are susceptible to insecticides (*c*_*SS*_ = 0.8). Lines show different disadvantages experienced by heterozygotes, *f*_SR_ =0 (blue), 0.5 (green) and 1 (red). **c**, **d** The size of the uninfected adult population relative to its value at equilibrium (black lines), the frequency of *Wolbachia* (red lines), and the frequency of the homozygote resistant genotype in infected mosquitoes (blue lines) over time. The grey shaded area shows the time period during which releases occur. Heterozygotes are fully susceptible to insecticides (*f*_SR_ =1). **c** Competition in the field population is intense (*C*_L_^*^ = 1); **d** Competition in the field population is low (*C*_L_^*^ = 0.018)

Releasing mosquitoes with this substantial fitness disadvantage requires much higher MRRs to meet the target *Wolbachia* frequency (Fig. 5a). The MRR is approximately an order of magnitude higher when heterozygotes are fully resistant (*f*_SR_ = 0) and even greater when heterozygotes are also disadvantaged (*f*_SR_ > 0). When heterozygotes are disadvantaged, the MRR is reduced less under more intense competition (Fig. 5a). This is because the *Wolbachia* benefits greatly from the introgression of resistant alleles through the population following releases, which causes a rise in the average fitness of its hosts. Introgression is slower when larval development periods are lengthened by more intense competition, which inhibits *Wolbachia* spread, particularly when heterozygotes are disadvantaged.

The time taken for the *Wolbachia* to become established, *T*_*E*_, is more strongly affected by density-dependent competition when released mosquitoes have a fitness disadvantage, particularly if heterozygotes are also affected (Fig. 5b). When heterozygotes are fully susceptible to insecticides (*f*_SR_ = 1), *T*_*E*_ is more than 3 years when competition in the field population is intense but less than a year when competition is low. This difference arises due to the strong effects on mosquito population dynamics of making large releases of *Wolbachia*-infected mosquitoes, particularly the reduction in the numbers of uninfected adults during the release period because of cytoplasmic incompatibility (Fig. 5c, d; black lines). The *Wolbachia* frequency drops rapidly to a low level after releases end due to the high fitness disadvantage (Fig. 5c, d; red lines), and cytoplasmic incompatibility therefore becomes less effective. If the population experiences low levels of density-independent mortality, *μ*_*I*_, it can recover rapidly from the suppression because the population growth rate is much higher at low densities. The population grows to almost pre-release levels before introgression of resistance allows the *Wolbachia* frequency to increase, and invasion is therefore very slow (Fig. 5c; black line). However, if density-independent mortality is high and population growth is less affected by density-dependence, the population is slow to recover from the reduction in density (Fig. 5d; black line). Introgression of resistance occurs while the population size remains low, allowing much faster *Wolbachia* invasion (Fig. 5d).

## Discussion

Our model of mosquito-*Wolbachia* dynamics, validated by observations from two independent field-cage *A. aegypti* populations, allows the combined effects of larval density-dependent variation in important mosquito fitness components to be characterized. We show how density-dependent competition can strongly affect the dynamics of *Wolbachia* field releases through complex interacting effects that are not represented in models assuming constant host demographic traits [6, 11]. First, the intensity of density-dependent competition in the field population is a very important determinant of the relative fitness of the well-nourished, insectary-reared insects that are released. Thus, when insects in the field experience intense competition, lower ratios of released to wild insects are required to achieve a given infection frequency. However, situations where competition is more intense are disadvantageous for field release strategies overall. We show that intense competition arises when population sizes are relatively high due to lower impacts of density-independent factors on mosquito survival. We predict that this leads to higher required absolute numbers of released mosquitoes. Further, larval development times increase under more intense competition, which slows *Wolbachia* spread by delaying increases in adult infection frequencies brought about by cytoplasmic incompatibility (see also [17]). *Wolbachia* releases can also cause strong perturbations in field population densities resulting in transient dynamics that are influenced by density-dependent population growth. We show that these effects can strongly affect the speed of *Wolbachia* invasion when released mosquitoes experience relative fitness disadvantages.

These findings demonstrate the importance of accounting for density-dependent variation in host fitness when attempting to predict *Wolbachia* invasion dynamics. Our ability to estimate the intensity of density-dependent competition in field *A. aegypti* populations is limited by the uncertainty about mosquito mortality and the severity of density-independent effects [6, 17, 18, 21, 28]. To reflect this uncertainty, we explore mosquito-*Wolbachia* dynamics across varying degrees of competition intensity. Our model can assist in the design of field release strategies by predicting mosquito demography and *Wolbachia* invasion across this range of competition intensity. The computer code for implementing our model (gyp_sim.cpp) has been made available (see doi 10.6084/m9.figshare.3980472).

The effects of density-dependence on the dynamics of *Wolbachia* after field releases are stronger when released mosquitoes experience fitness disadvantages. Our analysis considers fitness disadvantages arising from genetic susceptibility to chemical insecticides, which had a potential role in the failure of *w*Mel to invade and establish in field release trials in Brazil [9]. The rapid decline in *w*Mel frequency following these field releases suggests strong fitness disadvantages. Field releases may commonly be impacted by insecticide susceptibility in released mosquitoes because resistance in wild *A. aegypti* populations is widespread [29]. Insecticides targeted at adults remain an important means of arbovirus control [30], and attempts to release resistant mosquitoes may generate public health concerns. The mechanisms identified in our analyses also apply to other fitness disadvantages that may result from the genetic background of released mosquitoes (for example, those due to adaptation to laboratory environments).

The strategy of using insecticides or other means to suppress wild mosquito populations prior to releases has often been proposed as a means to facilitate *Wolbachia* invasion. However, several studies have expressed concern that a density-dependent increase in mosquito fitness may impede population suppression strategies [15, 18]. Our model predicts that increasing density-independent mortality reduces adult numbers despite alleviating the effects of competition on mosquito fitness, and therefore suggests that interventions to suppress populations by killing juveniles and adults are likely to be helpful. Our results also suggest that interventions that increase mosquito mortality rates will increase the rate of *Wolbachia* spread by reducing the effects of density-dependence that act to slow spread. However, this needs to be interpreted in the context of the method of population suppression. If chemical insecticides are employed, this could disadvantage the infected mosquitoes, which we have shown can substantially impede *Wolbachia* invasion.

Our Bayesian statistical models estimate variation in demographic traits over time for both *Wolbachia-*infected and uninfected mosquitoes in our experimental populations. The fitness of infected and uninfected mosquitoes did not differ significantly, except for the development times of larvae in the initial cohorts of one of the two experimental populations. However, these effects were small relative to the effects of larval density on development times and do not affect the conclusions of our field release simulations. We cannot assess whether *Wolbachia* infection caused these differences in larval development rate because our experiments do not control for effects of genetic differences between infected and uninfected mosquitoes. Experimentally controlling for effects of genetic variation is limited by the difficulty of creating genetically identical infected and uninfected mosquito lines [3]. Further, in field populations of *A. aegypti*, mtDNA variation remains tightly associated with *w*Mel infection [31], meaning that any mitochondrial genetic differences between released and field mosquitoes will persist following the releases.

Our methods may be applied to investigate the dynamics of other *Wolbachia* strains with potential to assist in the control of diseases transmitted by *A. aegypti*, for example the “life-shortening” *w*MelPop strain [32] and the *w*Mel*w*AlbB superinfection [33]. *A. aegypti* carrying these infections are strongly refractory to dengue [2, 33], but we know very little about the relative fitness of infected mosquitoes developing in the field and subject to food limitation (but see Ross et al. [34]). Even in the laboratory the *w*MelPop strain imposes high fitness costs on *A. aegypti* [3], and field releases have failed to establish *w*MelPop in wild populations [35].

We provide a framework for incorporating the effects of density-dependence into models of *A. aegypti* population dynamics on which further work can be based. More detailed models will need to explore the consequences of spatial structure and fluctuations in population size that can arise from stochastic processes and seasonality. Models based on ours but tailored to particular field settings could also be important in developing specific release strategies at different target locations.

## Conclusions

Our models incorporating larval density-dependent variation in mosquito demographic traits are effective in explaining mosquito and *Wolbachia* dynamics in field-cage *A. aegypti* populations. Our model simulations show qualitatively different host-symbiont dynamics compared to models that assume constant host demographic traits, with significant implications for *Wolbachia* field releases. Specifically, the intensity of density-dependent competition in the field population strongly affects the relative fitness of released insects, and is therefore important to predicting the infection frequency that will be achieved by a set program of releases. The development rate of juvenile mosquitoes is also strongly density-dependent, which affects the speed of *Wolbachia* spread. Further, transient *Wolbachia* dynamics associated with field releases are influenced by density-dependent mosquito population growth rates. These effects have greater impacts on dynamics when released mosquitoes experience fitness disadvantages. By incorporating these important ecological aspects of *Wolbachia*-host dynamics, our models can assist in understanding and achieving *Wolbachia* invasion in field mosquito populations.

## Methods

### Observations of *A. aegypti–Wolbachia* dynamics

We studied two populations of *A. aegypti* housed in field-cages receiving limited larval food resources, including the population experiment described in Hancock et al. [17] and a new population experiment presented in this study. The field-cage, of dimensions 7 × 4 × 5 m, was designed to simulate the natural habitat of *A. aegypti* in north-east Australia [36]. The first population (Population A) was initiated from a cohort of 100 pupae that were produced from larvae hatched on December 20, 2013, from wild caught eggs of an *A. aegypti* population located in Cairns, north-east Australia. All individuals were uninfected with *Wolbachia* [17]. The second population (Population B) was initiated from a cohort of 100 pupae produced from larvae hatched on October 17, 2014. A fraction (40 %) of these individuals was infected with *w*Mel. The uninfected pupae in this initial cohort were the third generation progeny of wild-caught eggs of an *A. aegypti* population located in Babinda, north-east Australia. The infected pupae were the progeny of a field-cage colony that has fixed *w*Mel infection and is regularly backcrossed with wild-caught *A. aegypti* from north-east Australia [3].

Both Populations A and B were maintained and monitored following the procedures described previously [17] (and also in Additional file 2: Text S1) for periods of 194 days (Population A) and 170 days (Population B). Regular collections were made (three times per week) of all eggs present in the field-cage. Eggs from each collection were transferred to a controlled temperature room at 26 °C where, after a 2 day incubation period, they hatched and all resulting first instar larvae were counted. A sample of 30 newly-hatched larvae was retained and the remaining individuals in the cohort were placed in the field-cage larval container habitat. A sample of 20 % of the pupae that eclosed in the field-cage populations on each day was retained (see [17]).

As described by Hancock et al. [17], Population A began receiving regular introductions (three times per week) of 16 *w*Mel-infected pupae at about 2 months (68 days) after its initiation. These infected pupae were taken from the field-cage colony described in Walker et al. [3]. The pupae were previously reared as larvae under the same food supply regime as that delivered to our field-cage experimental populations and experienced similar larval densities (see [17] for details of the larval rearing environment).

For both populations, we monitored the *Wolbachia* frequency over time by testing all individuals in the samples of first instar larvae and pupae for *Wolbachia* infection using real time PCR [37]. The *Wolbachia* frequency in each sample of first instar larvae was used to estimate the *Wolbachia* frequency in each cohort of newly-hatched first instar larvae. The *Wolbachia* frequency in the eclosed pupae was estimated by the frequency in the sampled pupae pooled over each week in order to reduce the sampling error.

The experimental design for Population B was informed by the results of the Population A experiment [17]. Specifically, in order to parameterize our population dynamic model (Fig. 1), we aimed to produce a wide range of *Wolbachia* frequencies in the eclosed pupae across the two populations. We therefore chose the *Wolbachia* frequency in the initial cohort of Population B to be close to the maximum frequency of 36 % observed in the eclosed pupae in Population A. Further, in Population A, we observed strong variation in per-capita female fecundity associated with changing larval density in the field-cage [17]. Therefore, in order to simplify the estimation of larval density-dependent variation in per-capita female fecundity, we did not make any introductions of externally-reared infected or uninfected mosquitoes into Population B following its initiation (see below and Additional file 2: Text S1).

### Estimating density-dependent demographic traits

We focus on two mosquito demographic traits, namely larval development time and per-capita adult female fecundity [17]. We express these demographic variables as functions of larval density, defining separate relationships for the infected and uninfected subpopulations, and infer the form of these functions using (1) our observed abundances of the juvenile mosquito life stages and (2) our observed *Wolbachia* infection frequencies in first instar larvae and pupae in our field-cage *A. aegypti* population over time (Additional file 2: Text S1). We make three assumptions, namely that (1) the sex ratio in the mosquito population is equal, (2) the population is panmictic (random mating) [38], and (3) adult females mate once only within a day of emergence [39]. Model parameters and values are defined in Additional file 1: Table S1.1.

#### Larval development time

For the *Wolbachia*-infected and uninfected individuals within each cohort of larvae we estimated the distributions of the probability of pupation over time [17]. We denote *p*_*i,c,W*_ and *p*_*i,c,U*_ as the probabilities that the infected and uninfected larvae within a cohort that hatch on day *c* pupate on day *i.* We assume that *p*_*i,c,W*_ and *p*_*i,c,U*_ follow gamma distributions with means *μ*_*c,W*_ and *μ*_*c,U*_, and standard deviations *σ*_*c,W*_ and *σ*_*c,U*_, respectively. We use flexible power law functions of larval density to estimate the means and standard deviations and choose a measure of average larval density that allows forward prediction (Additional file 2: Text S1). For the infected larvae hatched in cohort *c:*

2 $$ \begin{array}{c}\hfill {\mu}_{c,W}\left({\overline{L}}_c^P\right)={\alpha}_W+{\beta}_W{\left({\overline{L}}_c^P\right)}^{\gamma_W}\hfill \\ {}\hfill {\sigma}_{c,W}\left({\overline{L}}_c^P\right)={v}_W+{\eta}_W{\left({\overline{L}}_c^P\right)}^{\psi_W}\hfill \end{array} $$

where ${\overline{L}}_c^P$ is the estimated average larval density that the larvae in cohort *c* experience during the time period from hatching to eclosion of the first pupa from the cohort and *α*_*W*_, *β*_*W*_, *γ*_*W*_, *v*_*W*_, *η*_*W*_ and *ψ*_*W*_ are constant parameters. Functions of the same form as (2) but with different constant parameters (distinguished by subscript *U* instead of *W*) are used to estimate *μ*_*c,U*_ and *σ*_*c,U*_.

We estimate the 12 constant parameters using a Bayesian MCMC model that calculates the likelihood of observing our daily pupal eclosions and our *Wolbachia* infection frequencies in the samples of pupae (see Additional file 2: Text S1 for further details). The likelihood is also informed by our data on the number of first instar larvae hatched in each cohort, the daily larval survival (estimated using our larval counts; see Additional file 9: Figure S5.1, Additional file 10: Figure S5.2 and Additional file 2: Text S1), and the *Wolbachia* infection frequency in the first instar larvae sampled from each cohort. The priors are truncated uniform distributions that restrict the parameters’ ranges. Convergence diagnostics are presented in Additional file 11: Figure S7.1.

#### Per-capita female fecundity

We assume that only adult females with compatible matings (unaffected by cytoplasmic incompatibility) who have passed the minimum age for the first oviposition (*T*_*G*_; Additional file 1: Table S1.1) are capable of producing offspring. We require estimates of the abundance of these reproducing adult females (both *Wolbachia-*infected and uninfected) over time. We did not observe adult abundance or infection status in our study populations so we estimated these quantities using our daily records of pupal eclosion and observed *Wolbachia* frequencies in the sampled pupae (calculations are presented in Additional file 2: Text S1). This required estimates of the time required for pupal development, *T*_*P*_, the time lag between oviposition and hatching of eggs, *T*_*H*_, and the daily rate of pupal and adult survival *μ*_*A*_. We estimated *T*_*G*_, *T*_*P*_ and *T*_*H*_ directly from our data, and *μ*_*A*_ from Walker et al. [3] (Additional file 1: Table S1.1). Our methodology also assumes that maternal transmission of *Wolbachia* is perfect [3].

We define the per-capita fecundity of infected adult females on day *i, λ*_*i,W*_, as the number of infected first instar larvae hatched on that day per infected adult female capable of contributing offspring to this cohort. The per-capita fecundity of uninfected adults on day *i*, *λ*_*i,U*_, is similarly defined based on numbers of uninfected larvae hatched on day *i*. We assume that per-capita female fecundity is a log-linear function of the average larval density ${\overline{L}}_n^A$ over a fixed time lag of 3 weeks ending on day *n* = *i-T*_*G*_ *-T*_*P*_ (Additional file 2: Text S1). *λ*_*i,W*_ is estimated by:

3 $$ {\lambda}_{i,W}\left({\overline{L}}_n^A\right)={a}_W+{b}_W \log \left({\overline{L}}_n^A\right) $$

where *a*_*W*_ and *b*_*W*_ are constant parameters, and *λ*_*i,U*_ is estimated by a function of the same form as (3) but with different constant parameters, distinguished by substituting subscript *U* for subscript *W.*

We estimate the constant parameters using a Bayesian MCMC model that calculates the likelihood of our observed numbers of larvae hatched in each cohort and observed *Wolbachia* infection frequencies in each sample of newly hatched larvae (Additional file 2: Text S1 and Additional file 12: Figure S7.2). The likelihood is also informed by the daily counts of eclosed pupae and the *Wolbachia* infection frequency in the pupae sampled over each week. For Population A, we estimated the per-capita fecundity of uninfected, but not infected, females (*λ*_*i,U*_ only; see Additional file 2: Text S1) because the infected females that were introduced into the population were produced from larvae that had been reared separately [17].

### Modelling mosquito–*Wolbachia* dynamics

Our model of mosquito–*Wolbachia* dynamics assumes that larval development times and per-capita female fecundity depend on larval density (Fig. 1; double red lines) according to the forms estimated (Fig. 2a–c). We use the set of parameters that gives the maximum posterior probability for Population B. Mosquito demographic traits that are assumed to be temporally constant (Fig. 1; black solid lines) include the daily larval mortality, *μ*_*L*_, the daily pupal and adult mortality *μ*_*A*_, the time lags *T*_*G*_, *T*_*P*_ and *T*_*H*_ defined above, the strength of cytoplasmic incompatibility, *s*_*h*_, and the rate of maternal transmission of *Wolbachia*, 1− *ω* [3]. The mathematical model formulation and examples of the predicted dynamics are provided in Additional file 2: Text S1 and Additional file 13: Figure S4.1. The parameter values used in our model are provided in Additional file 1: Table S1.1.

#### Genetic fitness disadvantages in released mosquitoes

We use a simple representation of insecticide resistance in the mosquito population that assumes that resistance is determined at a single nuclear locus with susceptible (S), or resistant (R), alleles [40]. Frequencies of homozygotes (SS and RR) and heterozygotes (SR) in the juvenile offspring (eggs) produced by the (panmictic) adult population are determined by Mendelian inheritance (see Additional file 2: Text S1 for details of the mathematical model formulation). We assume that heterozygotes may experience an intermediate level of resistance [27, 40]. Insecticides are assumed to affect only adult mortality (and no other demographic traits). The frequency of the S-allele in the population declines over time after the end of the *Wolbachia* releases due to selection for the resistant phenotype.

## Additional files

Additional file 1: Table S1.1. Definitions and values of model parameters. (PDF 111 kb)
Additional file 2:
**Text S1.** Additional methods and results. (PDF 1746 kb)
Additional file 3: Figure S1.1. Observed mosquito and *w*Mel *Wolbachia* dynamics and the posterior fitted values. Red lines show the Markov Chain Monte Carlo iteration with the highest posterior probability and blue shaded areas show the 95 % credible interval. Results for Populations A and B are on the left and right of the dashed vertical line respectively. (A) Red circles show the observed *w*Mel frequency in the pupae that eclosed in each week and vertical lines are the exact binomial 95 % confidence intervals. (B) Black circles show the observed weekly average pupal eclosion (PDF). (PDF 108 kb)
Additional file 4: Figure S1.2. The observed cumulative number of pupae (red lines) and hatched larvae (black lines) compared and the posterior fitted values. The predicted cumulative numbers of pupae (blue lines) and hatched larvae (green lines) are given by the maximum posterior probability iteration. Blue and green shaded areas show the 95 % credible interval for the cumulative numbers of pupae and hatched larvae respectively. Results for Populations A and B are shown on the left and right side of the vertical dotted line. (PDF 65 kb)
Additional file 5: Figure S1.3. Observed numbers of larvae hatched and the posterior fitted values. Red lines show the Markov Chain Monte Carlo iteration with the highest posterior probability and blue shaded areas show the 95 % credible interval. Results for Population A and B are on the left and right of the dashed vertical line, respectively. Black circles show the observed weekly average total number of larvae hatched. (PDF 62 kb)
Additional file 6: Figure S2.1. Posterior distributions of the differences in means (*μ*_*c*,*U*_ − *μ*_*c*,*W*_; blue lines) and standard deviations (*σ*_*c,U*_ *− σ*_*c,W*_; red lines) of the development time distributions of infected and uninfected larvae hatched in each cohort. For Population A, results for cohorts 47, 48 and 52 are not shown because the observed infection frequency in these first instar larvae was close to 0. For Population B, results for cohorts 19, 22 and 26 are not shown because the observed infection frequency in these first instar larvae was close to 1. (PDF 238 kb)
Additional file 7: Figure S3.1. Observed *w*Mel *Wolbachia* dynamics in the first instar larvae and the posterior fitted values, for Population B. The red lines show the Markov Chain Monte Carlo iteration with the highest posterior probability and blue shaded area shows the 95 % credible interval. Red circles show the observed *w*Mel frequency in the first instar larvae that hatched in each week and vertical lines are the exact binomial 95 % confidence intervals. (PDF 61 kb)
Additional file 8: Figure S3.2. Posterior distributions of the difference in the predicted per-capita fecundity of *Wolbachia-*infected and uninfected adult females (*λ*_*i,U*_ − *λ*_*i,W*_) for each cohort for Population B. (PDF 119 kb)
Additional file 9: Figure S5.1. Total number of larvae counted in each week (black line and circles), divided into first and second instars (blue shading), third instars (yellow shading), and fourth instars (pink shading). A Population A; the red arrow indicates the day that introductions of *w*Mel-infected pupae were initiated and the black arrow indicates the day that both *w*Mel introductions and egg hatching were terminated (see [17]). B Population B; the black arrow indicates the day that egg hatching was terminated. (PDF 87 kb)
Additional file 10: Figure S5.2. The observed (black line and markers) and interpolated (red dashed line) larval survival over time. (PDF 57 kb)
Additional file 11: Figure S7.1. Gelman–Rubin plots of the posterior fitted values. Plots show the shrink factor for three chains each starting at different initial values: A, B. *β*_*U*_ and *β*_*W*_; C, D. *α*_*U*_ and *α*_*W*_; E, F. *γ*_*U*_ and *γ*_*W*_; G, H. *v*_*U*_ and *v*_*W*_; I, J. *η*_*U*_ and *η*_*W*_; K, L. *ψ*_*U*_ and *ψ*_*W*_. (PDF 199 kb)
Additional file 12: Figure S7.2. Gelman–Rubin plots showing the shrink factor for three chains each starting at different initial values: A. *b*; B. *a*. (PDF 71 kb)
Additional file 13: Figure S4.1. Long term behaviour of the uninfected mosquito population in the absence of *Wolbachia*. A, B. The intensity of density-dependent competition is low (*C*_*L*_^*^ = 0.018, *μ*_*I*_ = 0.12). C, D. The intensity of density-dependent competition is high (*C*_*L*_^*^ = 1.0, *μ*_*I*_ = 0). A, C. Numbers of larvae (black line) and adults (red line). B, D. Per-capita adult female fecundity (red lines), larval development time mean (blue line) and standard deviation (black line). (PDF 107 kb)
