# Supplementary material for: Predicting Wolbachia invasion dynamics in Aedes aegypti populations using models of density-dependent demographic traits
Source: BMC Biol. 2016 Nov 8;14:96. doi: 10.1186/s12915-016-0319-5 (PMC5100186; doi:10.1186/s12915-016-0319-5)
Supplement: Additional file 1: Table S1.1. — Definitions and values of model parameters. (PDF 111 kb) [file 12915_2016_319_MOESM1_ESM.pdf]

Predicting Wolbachia invasion dynamics in Aedes aegypti populations using models of density-dependent demographic traits

Penelope A. Hancock, Vanessa L. White, Scott A. Ritchie, Ary A. Hoffmann, H. Charles J. Godfray

BMC Biology 2016

Additional file 1: Table S1.1. Definitions and values of model parameters.

Symbol	Definition	Value		Source
T_P	Duration of the pupal stage	2 days		This study
T_G	Minimum time for females to become gravid following emergence	4 days		This study
T_H	Time between oviposition and hatching of eggs	6 days		This study
ω	The proportion of uninfected offspring produced by *Wolbachia*-infected females	0.01		(Walker *et al.* 2011)
s_h	The proportion of unviable offspring from an incompatible mating	0.99		(Walker *et al.* 2011)
		Semi-field cage population	*Field release simulations*	
$(\alpha_U,\beta_U,\gamma_U)$; $(\alpha_W,\beta_W,\gamma_W)$	Parameters of functions describing mean larval development time	(0.68, 0.16, 0.68)[a]; (2.2, 0.013, 0.98)[a]; (1.8, 0.54, 0.53)[b]; (4.5, 0.22, 0.64)[b];	(1.8, 0.54, 0.53); (1.8, 0.54, 0.53);	This study
(ν_U,η_U,ψ_U); (ν_W,η_W,ψ_W)	Parameters of functions describing the standard deviation of larval development time	(8.3, 0.81, 0.33)[a]; (4.1, 1.7, 0.27)[a]; (0.22, 0.017, 0.87)[b]; (4.8, 0.029, 0.80)[b];	(0.22, 0.017, 0.87); (0.22, 0.017, 0.87)	This study
a_U,b_U; a_W,b_W	Parameters of functions describing per-capita female fecundity	(29.5, -3.36)[a]; (28.5, -3.25)[b]; (29.1, -3.35)[b];	(28.5, -3.25); (28.5, -3.25);	This study
μ_L^c	Daily larval mortality	0.25-0.01	0.05	This study
μ_A^c	Daily pupal and adult mortality	0.03	0.03	(Walker *et al.* 2011)
$\lambda_{\min}, \mu_{\max}, \sigma_{\max}$	Lower limit on per-capita female fecundity, and upper limits on larval development time means and standard deviations	0.5, 60 days, 40 days	0.5, 60 days, 40 days	This study

a,b Value corresponding to the MCMC iteration with the highest posterior probability when observations from Population A (a) and Population B (b) are used to inform the Bayesian MCMC model (see text)
