# Supplementary material for: Predicting Wolbachia invasion dynamics in Aedes aegypti populations using models of density-dependent demographic traits
Source: BMC Biol. 2016 Nov 8;14:96. doi: 10.1186/s12915-016-0319-5 (PMC5100186; doi:10.1186/s12915-016-0319-5)
Supplement: Additional file 2: — Text S1. Additional methods and results. (PDF 1746 kb) [file 12915_2016_319_MOESM2_ESM.pdf]

Predicting Wolbachia invasion dynamics in Aedes aegypti populations using models of density-dependent demographic traits

Penelope A. Hancock, Vanessa L. White, Scott A. Ritchie, Ary A. Hoffmann, H. Charles J. Godfray

BMC Biology 2016

Additional file 2: Text S1. Additional Methods and Results

S1. Observed mosquito and *Wolbachia* dynamics compared to the model predictions ……2

S2. Differences in development times of *Wolbachia*-infected and uninfected larvae……2

S3. Differences in per-capita fecundity of *Wolbachia*-infected and uninfected adult females……3

S4. Mathematical model of mosquito-*Wolbachia* dynamics……3

S5. Semi-field experimental study of mosquito-*Wolbachia* dynamics……6

S6. Bayesian MCMC estimation of density-dependent mosquito demographic traits……7

S7. MCMC convergence results……10

S1. Observed mosquito and *Wolbachia* dynamics compared to the model predictions.

Larval development times

Our predicted relationships between larval development times and larval density for the infected and uninfected subpopulations describe the major features of the observed weekly *Wolbachia* infection frequencies in the pupae (Additional file3: Fig. S1.1A) and the observed average weekly pupal eclosions (Additional file 3: Fig. S1.1B) for both populations. The model over-estimates the *Wolbachia* frequency in weeks 11-13 for Population B and over-estimates pupal eclosion in weeks 17-20 for Population A. These models of larval development times accurately explain the cumulative production of pupae over time for both populations (Additional file 4: Fig. S1.2).

Per-capita female fecundity

For Population A our predicted relationship between larval density and the per-capita fecundity of uninfected females accurately describes the dynamics of the numbers of uninfected larvae hatched (Additional file 5: Fig. S1.3; Population A). For Population B, our predicted relationship between larval density and the per-capita fecundity of (infected and uninfected) females describes the major features of the dynamics of the total numbers of hatched larvae except for a drop and rise in hatch size in the last four weeks of larval recruitment (weeks 11-15) that is not captured by the model (Additional file 5: Fig. S1.3; Population B). These models accurately explain the cumulative number of larvae hatched over time for both populations (Additional file 4: Fig. S1.2).

S2. Differences in development times of *Wolbachia*-infected and uninfected larvae

We determine whether there are credible differences between the predicted mean development times of the infected and uninfected larvae in each cohort by calculating the 95% credible interval (CI) of the differences in means for each draw from the posterior distribution (based on 250000 draws; see section S8). We use the same method to assess credible differences in the predicted development time standard deviations for the infected and uninfected larvae in each cohort.

For Population A, infected larvae were only present in cohorts hatched after week 11 (cohorts 28-54). For Population A, the 95% CI of the posterior distribution of the differences in mean development times contains zero for all cohorts (Additional file 6: Fig. S2.1; Population A (blue lines)). The 95% CI of the posterior distribution of the differences in the standard deviations also contains zero for all cohorts (Additional file 6: Fig. S2.1; Population A (red lines)).

For Population B the 95% CI of the posterior distribution of the differences in mean development times is greater than zero for all cohorts hatched in weeks 1-5 (Additional file 6: Fig. S2.1; Population B (cohorts 1-15, blue lines)). Faster development of infected larvae in these earlier cohorts resulted in a high *w*Mel frequency in the first week following pupation of the first individual (Additional file 3: Fig. S1.1A; the observed frequency is 1.0 in week 6 for Population B)). The predicted development times of infected larvae are more variable than those of uninfected larvae (Fig. 3B), but this trend is not strongly supported because the

95% CI of the posterior distribution of the differences in the standard deviations is close to zero for all cohorts (Additional file 6: Fig. S2.1; Population B (red lines)).

S3. Differences in per-capita fecundity of *Wolbachia*-infected and uninfected adult females

For Population B, the fitted models of per-capita fecundity of infected and uninfected adult females as functions of larval density describe the major features of the *w*Mel frequency dynamics in the first instar larvae (Additional file 7: Fig. S3.1). We determine whether there are credible differences between the predicted per-capita fecundity of the infected and uninfected females by calculating the 95% credible interval (CI) of the differences in per-capita female fecundity for each draw from the posterior distribution (based on 400000 draws; see Additional file 1: section S8). Predicted differences in per-capita female fecundity between infected and uninfected females were clearly not significant (Additional file 8: Fig. S3.2).

S4. Mathematical model of mosquito-*Wolbachia* dynamics

We develop a stage-structured model to describe the dynamics of both the mosquito population and *Wolbachia* infection (Fig. 1). Definitions and values of the model parameters are provided in Additional file 1: Table S1.1. The dynamics of the larval, pupal and adult stages are described by the following equations:

Larvae

For each day *i*, we define $\lambda_{i,W}$ and $\lambda_{i,U}$ as the per-capita fecundity of adult females that are old enough to produce eggs. For uninfected mosquitoes we consider only adult females with compatible matings (unaffected by CI; see Methods and section S6). As described in the Methods, $\lambda_{i,W}$ and $\lambda_{i,U}$ are modeled as functions of the larval density averaged over a fixed time lag. Females infected with *Wolbachia* transmit the bacteria to a fraction $1-\omega$ of their offspring. Thus, the numbers of *Wolbachia*-infected and uninfected first instar larvae that are hatched on day *c,* $H_{c,W}$ and $H_{c,U}$, are given by:

$$H_{c,U} = \lambda_{c-T_H,U}\tilde{A}_{c-T_H,U} + \omega\lambda_{c-T_H,W}\tilde{A}_{c-T_H,W}$$
$$H_{c,W} = \lambda_{c-T_H,W}(1-\omega)\tilde{A}_{i-T_H,W} \quad \text{(S4.1)}$$

where $\tilde{A}_{i,W}$ and $\tilde{A}_{i,U}$ are the numbers of infected and uninfected adult females capable of contributing offspring on day *i* (defined in below in eqns S4.4).

Pupae

The number of infected larvae in a cohort hatched on day *c* that eclose as pupae on day *i*, $P_{i,W,c}$, was estimated by:

$$P_{i,W,c} = H_{c,W}\prod_{k=c+1}^{i}(1-\mu_{L,k})p_{k,c,W} \quad \text{(S4.2)}$$

The number of uninfected larvae hatched in cohort *c* that eclose as pupae on day *i*, $P_{i,U,c}$, is given by the same equation with subscript *U* substituted for subscript

W. As discussed in the main text, the probabilities of pupation, $p_{i,c,W}$ and $p_{i,c,U}$ are modeled as functions of the average larval density, $\bar{L}_c^P$ (see Methods and section S6).

Adults

The numbers of infected and uninfected adults in the population on day *i*, $A_{i,W}$ and $A_{i,U}$, are given by:

$$A_{i,W} = \sum_{k=0}^{i-T_P} P_{k,W}(1-\mu_A)^{i-k-1}$$

$$A_{i,U} = \sum_{k=0}^{i-T_P} P_{k,U}(1-\mu_A)^{i-k-1}, \quad i \geq T_P \qquad \text{(S4.3)}$$

The numbers of infected and uninfected adult females capable of contributing offspring on day *i* (see Methods) are given by:

$$\tilde{A}_{i,W} = 0.5 \sum_{k=0}^{i-T_P-T_G-T_H} P_{k,W}(1-\mu_A)^{i-k-1}$$

$$\tilde{A}_{i,U} = 0.5 \sum_{k=0}^{i-T_P-T_G-T_H} P_{k,U}(1-\mu_A)^{i-k-1}(1-s_h f_{k+T_P}^A), \quad i \geq T_P + T_G + T_H \qquad \text{(S4.4)}$$

Equilibria in the absence of Wolbachia

Assume *Wolbachia* is not present in the mosquito population and let $\tilde{A}^*$ denote the equilibrium number of adult females old enough to produce offspring, and P^* and H^* denote the equilibrium numbers of pupae that eclose and larvae that hatch on each day, respectively. Then define the equilibrium per-capita female fecundity, λ^*, and the equilibrium probability that a larvae pupates at age *a*, p_a^*. Then,

$$H^* = \lambda^* \tilde{A}^* \qquad \text{(S4.5)}$$

$$P^* = H^* \sum_{a=0}^{a_{\max}^L} \prod_{k=0}^{a} (1-\mu_L)^{k-1} p_k^* \qquad \text{(S4.6)}$$

where larvae are assumed to live no longer than $a_{\max}^L$. We then define the total survival through the juvenile and pre-mature adults stages (including the larval and pupal stages and the early part of the adult stage when females are too young to produce offspring), θ_J^*, given by:

$$\theta_J^* = \sum_{a=0}^{a_{\max}^L} \prod_{k=0}^{a} (1-\mu_L)^{k-1} p_k^* \theta_P \theta_{A_G} \qquad \text{(S4.7)}$$

where θ_P and θ_{A_G} are the probabilities of surviving through the pupal and immature adult stages respectively. Then,

$$A^* = P^* \theta_P \theta_{A_G} \sum_{j=1}^{\infty} (1-\mu_A)^{j-1}$$

$$= \frac{\lambda^* A^* \theta_J^*}{\mu_A} \qquad \text{(S4.8)}$$

and

$$\frac{\lambda^{*}\theta_{J}^{*}}{\mu_{A}}=1 \qquad \text{(S4.9)}$$

Modelling genetic susceptibility to insecticides

According to a two-allele model of insecticide resistance, we define three genotypes: homozygote susceptible, heterozygote and homozygote resistant. We denote these genotypes by subscripts SS, SR and RR respectively. We categorize the infected and uninfected mosquitoes in the population according to these genotypes, giving six classes. We denote the frequencies of homozygote susceptible, heterozygote and homozygote resistant adults in the total population on day *i* as q_i^{SS}, q_i^{SR} and q_i^{RR}. If we assume for simplicity that cytoplasmic incompatibility is complete ($s_h = 1$) and maternal transmission of *Wolbachia* is perfect ($\omega = 0$) then the numbers of *Wolbachia*-infected offspring hatched on day *c* of each genotype are given by:

$$H_{c,W}^{SS}=0.5\lambda_{c-T_H,W}((q_{i-T_P-T_G}^{SS}+0.5q_{i-T_P-T_G}^{SR})A_{i-T_P-T_G-T_H,W}^{SS}(1-\mu_A^{SS})^{i-T_P-T_G-1}+$$
$$(0.5q_{i-T_P-T_G}^{SS}+0.25q_{i-T_P-T_G}^{SR})A_{i-T_P-T_G-T_H,W}^{SR}(1-\mu_A^{SR})^{i-T_P-T_G-1})$$

$$H_{c,W}^{SR}=0.5\lambda_{c-T_H,W}((q_{i-T_P-T_G}^{SS}+0.5q_{i-T_P-T_G}^{SR})A_{i-T_P-T_G-T_H,W}^{RR}(1-\mu_A^{RR})^{i-T_P-T_G-1}+$$
$$(0.5q_{i-T_P-T_G}^{SS}+0.5q_{i-T_P-T_G}^{SR}+0.5q_{i-T_P-T_G}^{SS})A_{i-T_P-T_G-T_H,W}^{SR}(1-\mu_A^{SR})^{i-T_P-T_G-1}+$$
$$(q_{i-T_P-T_G}^{RR}+0.5q_{i-T_P-T_G}^{SR})A_{i-T_P-T_G-T_H,W}^{SS}(1-\mu_A^{SS})^{i-T_P-T_G-1})$$

$$H_{c,W}^{RR}=0.5\lambda_{c-T_H,W}((q_{i-T_P-T_G}^{RR}+0.5q_{i-T_P-T_G}^{SR})A_{i-T_P-T_G-T_H,W}^{RR}(1-\mu_A^{RR})^{i-T_P-T_G-1}+$$
$$(0.5q_{i-T_P-T_G}^{RR}+0.25q_{i-T_P-T_G}^{SR})A_{i-T_P-T_G-T_H,W}^{SR}(1-\mu_A^{SR})^{i-T_P-T_G-1})$$

(S4.10)

Uninfected females can only mate successfully with uninfected males, so we define the adult gene frequencies in the uninfected subpopulation as $q_{i,U}^{SS}, q_{i,U}^{SR}$ and $q_{i,U}^{RR}$. Then, the numbers of uninfected offspring hatched on day *c* of each genotype are:

$$H_{c,U}^{SS}=0.5(1-f_{i-T_P-T_G}^{A})\lambda_{c-T_H,U}((q_{i-T_P-T_G,U}^{SS}+0.5q_{i-T_P-T_G,U}^{SR})A_{i-T_P-T_G-T_H,U}^{SS}(1-\mu_A^{SS})^{i-T_P-T_G-1}+$$
$$(0.5q_{i-T_P-T_G,U}^{SS}+0.25q_{i-T_P-T_G,U}^{SR})A_{i-T_P-T_G-T_H,U}^{SR}(1-\mu_A^{SR})^{i-T_P-T_G-1})$$

$$H_{c,U}^{SR}=0.5(1-f_{i-T_P-T_G}^{A})\lambda_{c-T_H,U}((q_{i-T_P-T_G,U}^{SS}+0.5q_{i-T_P-T_G,U}^{SR})A_{i-T_P-T_G-T_H,U}^{RR}(1-\mu_A^{RR})^{i-T_P-T_G-1}+$$
$$(0.5q_{i-T_P-T_G,U}^{SS}+0.5q_{i-T_P-T_G,U}^{SR}+0.5q_{i-T_P-T_G,U}^{SS})A_{i-T_P-T_G-T_H,U}^{SR}(1-\mu_A^{SR})^{i-T_P-T_G-1}+$$
$$(q_{i-T_P-T_G,U}^{RR}+0.5q_{i-T_P-T_G,U}^{SR})A_{i-T_P-T_G-T_H,U}^{SS}(1-\mu_A^{SS})^{i-T_P-T_G-1})$$

$$H_{c,U}^{RR}=0.5\lambda_{c-T_H,U}(1-f_{i-T_P-T_G}^{A})((q_{i-T_P-T_G,U}^{RR}+0.5q_{i-T_P-T_G,U}^{SR})A_{i-T_P-T_G-T_H,U}^{RR}(1-\mu_A^{RR})^{i-T_P-T_G-1}+$$
$$(0.5q_{i-T_P-T_G,U}^{RR}+0.25q_{i-T_P-T_G,U}^{SR})A_{i-T_P-T_G-T_H,U}^{SR}(1-\mu_A^{SR})^{i-T_P-T_G-1}) \qquad \text{(S4.11)}$$

Predicted dynamics of the mosquito population in the absence of Wolbachia *releases.*

When larval densities exceeded those that we observed (>5000; Additional file 9: Fig. S5.1), our predicted values of the density-dependent demographic rates (per-capita adult female fecundity and larval development time means and standard deviations) reached extreme values that we consider unlikely to occur in real populations. Therefore we set maximum and minimum limits on the values of these density-dependent demographic rates (Additional file 1: Table S1.1). These values are close to the limits estimated from our data (Fig. 2A-C).
For all sets of parameters and initial conditions that we explored (see Results and Additional file 1: Table S1.1), numerical simulations of our model showed a single non-zero equilibrium that displayed stable fluctuations, defining the carrying capacity of the mosquito population (e.g Additional file 13: Fig. S4.1). The number of larvae and adults at carrying capacity was estimated by the mean value obtained from the final year of the simulation, by which time the population had reached equilibrium (e.g. Additional file 13: Fig. S4.1).

S5. Semi-field experimental study of mosquito-*Wolbachia* dynamics
Both populations were maintained and monitored following the procedures described in (Hancock *et al.* 2016). In brief, adult females were allowed to feed on blood from a live human three times a week. A larval habitat consisted of a single container (a 5L bucket) filled with 2L of water. The larval container received a fixed amount of food (0.32g ground lucerne) three times per week. The larval container was lined with flannel cloth strips on which adult females oviposited eggs. All eggs that had been oviposited on the strips were removed from the cage three times a week, placed in an incubator at 26°C for two days, then stimulated to hatch. All newly-hatched larvae in each cohort were counted, a sample of 30 was retained, and the remaining individuals were placed back in the semi-field cage larval container. *A. aegypti* population dynamics were monitored by daily counts of all newly eclosed pupae and counts three times a week of all larvae (categorized as first, second, third or fourth instar) (Additional file 9: Fig. S5.1). A sample of 20% of the pupae that eclosed on each day was retained. We ceased adding newly-hatched larvae to the population after day 147 (week 21) for Population A and after day 108 (week 16) for Population B, and the remaining individuals either matured or died.

Estimating daily larval survival
The larval survival from day *j* to day *i*, s_{ji}, can be calculated at frequent intervals using the three weekly counts of the total number of larvae, L_j, and the daily counts of the number of eclosed pupae, P_i (Additional file 1: Table S1.1):

$$s_{ji} = \frac{L_i + \sum_{k=j+1}^{i} P_k}{L_j} \qquad \text{(S6.1)}$$

The s_{ji} were interpolated to estimate the daily larval survival values between all days *k-1* to *k*, s_k, as follows:

$$s_k = \left(s_{ij}\right)^{1/(i-j)} \qquad \text{(S6.2)}$$

The observed and interpolated survival values are shown in Additional file 10: Figure S5.2.

S6. Bayesian MCMC estimation of density-dependent mosquito demographic traits

We use Bayesian Markov Chain Monte Carlo (MCMC) methods to estimate the parameters of functions predicting mosquito demographic rates by variation in larval density (eqns 2 & 3). We focus on two mosquito demographic rates: the larval pupation probability and the per-capita adult female fecundity. We derive separate estimates of these demographic rates for the *Wolbachia*-infected and uninfected subpopulations. We use different measures of average larval density to predict each of the demographic processes. To predict the probability of pupation we use the estimated average larval density that the larvae in cohort *c* experience during the time period from hatching to eclosion of the first pupa from the cohort, $\bar{L}_c^P$. We use the time average of the larval density over a fixed time lag ending on day $n=i-T_G-T_P$, $\bar{L}_n^A$, to predict per-capita female fecundity. We set this time lag to three weeks (Hancock *et al.* 2016).

(i) Estimating larval development time distributions

The pupation probabilities of the infected and uninfected larvae in each cohort *c*, $p_{i,c,W}$ and $p_{i,c,U}$, are modeled as gamma distributions where the means and standard deviations are power law functions of $\bar{L}_c^P$ (eqn 2). The average larval density that larvae in cohort *c* experience during the time period from hatching to eclosion of the first pupa from the cohort, $\bar{L}_c^P$, is calculated as:

$$\bar{L}_c^P = \sum_{j=c}^{T_c} L_j / (T_c - c) \qquad \text{(S6.1)}$$

where L_j is the total larval density on day *j* and T_c is the time that the first pupa from cohort *c* ecloses. This allows us to obtain an estimate of $\bar{L}_c^P$ that is fixed following the time that the first pupa from the cohort ecloses.

The mean number of pupae eclosing in week *i*, μ_{P_w} is estimated by

$$\mu_{P_w} = \left(\sum_{\forall c} P_{i,c,W} + P_{i,c,U} \right) / 7 \qquad \text{(S6.2)}$$

where $P_{i,c,W}$ and $P_{i,c,U}$ are the estimated numbers of infected and uninfected larvae in a cohort hatched on day *c* that emerge as pupae on day *i*. For the infected subpopulation:

$$P_{i,W,c} = H_{c,W} \prod_{k=c+1}^{i} (1-\mu_{L,k}) p_{k,c,W} \qquad \text{(S6.3)}$$

where $H_{c,W}$ is the number of *Wolbachia*-infected larvae hatched on day *c* and $\mu_{L,k}$ is the daily larval mortality on day *k* (see section S5). $P_{i,c,U}$ is estimated using the same equation as S6.3 with subscript *U* substituted for subscript *W.* The numbers of infected and uninfected larvae hatched on day *c* are estimated by $H_{c,W} = f_c^L H_c$ and $H_{c,U} = (1-f_c^L) H_c$ where is H_c the total number of larvae hatched

on day *c* and f_c^L is the observed *Wolbachia* frequency in the larvae sampled on day *c*.

The mean number of pupae observed in week *w,* P_w , is assumed to follow a normal distribution $N(\mu_{P_w},\sigma_P)$. The number of pupae uninfected with *Wolbachia,* $n_{w,U}^P$, observed in a sample of n_w^P pupae from week *w* has a binomial distribution $B(n_{w,U}^P,n_w^P,\bar{f}_w^P)$ where $\bar{f}_w^P$ is the expected *Wolbachia* frequency in the pupae that eclose in week *w.* The likelihood is given by:

$$p(\{n_{w,U}^P,n_{w,U}^P\},\{P_i\}\,|\,\{H_c,f_c^L\},\{L_i\},\Theta)=\prod_{\forall i}N(P_w,\mu_{P_w},\sigma_P)B(n_{w,U}^P,n_{w,W}^P,\bar{f}_w^P) \quad \text{(S6.4)}$$

where Θ is the vector of constant model parameters, $\Theta=[\alpha_U,\alpha_W,\beta_U,\beta_W,\gamma_U,\gamma_W,\nu_U,\nu_W,\eta_U,\eta_W,\psi_U,\psi_W,\sigma_P]$. The posterior distribution is proportional to the product of the likelihood (eqn S6.4) and the prior $p(\Theta)$. We use uniform prior distributions: $U(0,\infty)$ for α_W and α_U; $U(0,10)$ for β_W and β_U; $U(0.2,2.0)$ for γ_W and γ_U ; $U(0,5)$ for ν_W and ν_U ; $U(0,\infty)$ for η_W and η_U ; $U(0.2,2.0)$ for ψ_W and ψ_U ; $U(1,5)$ for σ_P . We found that the model fit to the observations for Population B was improved by setting $\sigma_P=1.0$ for pupae that eclose in the first 3 weeks. This reflects our belief that larval development times can be estimated with greater accuracy for these individuals because there are fewer larval cohorts that contribute to these pupae.

We use a Metropolis-Hastings Markov Chain Monte Carlo (MCMC) algorithm to sample from the posterior, with a fat-tailed student *t* distribution as the proposal distribution. For each parameter the *j*th proposal is given by:

$$\begin{aligned}
&\alpha_W^{(j)}=\alpha_W^{(j-1)}+c_1\varepsilon_1^{(j)}; \quad \alpha_U^{(j)}=\alpha_U^{(j-1)}+c_2\varepsilon_2^{(j)}\\
&\beta_W^{(j)}=\beta_W^{(j-1)}+c_3\varepsilon_3^{(j)}; \quad \beta_U^{(j)}=\beta_U^{(j-1)}+c_4\varepsilon_4^{(j)}\\
&\gamma_W^{(j)}=\gamma_W^{(j-1)}+c_5\varepsilon_5^{(j)}; \quad \gamma_U^{(j)}=\gamma_U^{(j-1)}+c_6\varepsilon_6^{(j)}\\
&\nu_W^{(j)}=\nu_W^{(j-1)}+c_7\varepsilon_7^{(j)}; \quad \nu_U^{(j)}=\nu_U^{(j-1)}+c_8\varepsilon_8^{(j)}\\
&\eta_W^{(j)}=\eta_W^{(j-1)}+c_9\varepsilon_9^{(j)}; \quad \eta_U^{(j)}=\eta_U^{(j-1)}+c_{10}\varepsilon_{10}^{(j)}\\
&\psi_W^{(j)}=\psi_W^{(j-1)}+c_{11}\varepsilon_{11}^{(j)}; \quad \psi_W^{(j)}=\psi_W^{(j-1)}+c_{12}\varepsilon_{12}^{(j)}\\
&\sigma_W^{(j)}=\sigma_W^{(j-1)}+c_{13}\varepsilon_{12}^{(j)}
\end{aligned} \quad \text{(S6.5)}$$

where $\varepsilon_1,....,\varepsilon_{12}$ are random variables drawn from a student *t* distribution with 0.8 degrees of freedom. For Population A c_1=4.0; c_2=4.0; c_3=0.01; c_4=0.01 ; c_5=0.01 ; c_6= 0.01; c_7= 6.0; c_8= 6.0; c_9= 0.125; c_{10}= 0.125; c_{11}= 0.04; c_{12}=0.04; c_{13}=1.0 and for Population B c_1=4.0; c_2=4.0; c_3=0.075; c_4=0.1 ; c_5=0.01 ; c_6= 0.01; c_7= 6.0; c_8= 6.0; c_9= 0.125; c_{10}= 0.125; c_{11}= 0.025; c_{12}=0.04; c_{13}=1.0.

For larval cohorts that were hatched within two weeks before the final hatch date we found that the model provided an improved fit to the observed numbers of eclosed pupae by multiplying $P_{i,c,U}$ and $P_{i,c,W}$ by a factor $F_c(i)=\sqrt{N-c/N-i}$ until all larvae in these cohorts had either pupated or died. We justify this assumption by the fact that larval densities decline rapidly after the final hatch date (Additional file 9: Fig. S5.1).

(ii) Estimating per-capita female fecundity

Cytoplasmic incompatibility (CI) affects the uninfected subpopulation when *Wolbachia* infection is present in the mosquito population, and the number of uninfected females that can reproduce normally is reduced in proportion to the *Wolbachia* frequency (under assumptions (i)-(iii) defined in the second section of the Methods). Thus the numbers of *Wolbachia*-infected and uninfected females capable of producing offspring that are present on day *i*, $\tilde{A}_{i,W}$ and $\tilde{A}_{i,U}$, are estimated by:

$$\tilde{A}_{i,W} = 0.5 \sum_{k=0}^{i-T_P-T_G} P_k (1-\mu_A)^{i-k-1} \bar{f}_w^P$$
$$\tilde{A}_{i,U} = 0.5 \sum_{k=0}^{i-T_P-T_G} P_k (1-\mu_A)^{i-k-1} (1-\bar{f}_w^P)(1-s_h f_{k+T_P}^A); \quad k \in w, \quad i \geq T_P + T_G \qquad \text{(S6.6)}$$

where P_k is the number of pupae that eclose on day *k*, $\bar{f}_w^P$ is the *Wolbachia* infection frequency in the pupae that eclose during week *w* (estimated from our data), s_h is the strength of CI (Additional file 1: Table S1.1) and f_k^A is the *Wolbachia* infection frequency in the adult mosquito population on day *k*. Calculation of f_k^A is based on estimation of the total numbers of infected and uninfected adults in population on day *i*, $A_{i,W}$ and $A_{i,U}$:

$$A_{i,W} = \sum_{k=0}^{i-T_P} P_k (1-\mu_A)^{i-k-1} \bar{f}_w^P$$
$$A_{i,U} = \sum_{k=0}^{i-T_P} P_k (1-\mu_A)^{i-k-1} (1-\bar{f}_w^P); \quad k \in w, \quad i \geq T_P \qquad \text{(S6.7)}$$

Then $f_i^A = A_{i,W} / (A_{i,W} + A_{i,U})$. The per-capita fecundity of infected and uninfected adults on day *i*, $\lambda_{i,U}$ and $\lambda_{i,U}$, is then:

$$\lambda_{i,W} = \frac{H_{c,W}}{\tilde{A}_{i,W}}; \quad \lambda_{i,U} = \frac{H_{c,U}}{\tilde{A}_{i,U}} \qquad \text{(S6.8)}$$

where $c = i + T_H$. As described in the Methods, $\lambda_{i,W}$ and $\lambda_{i,U}$ are modeled as functions of $\bar{L}_n^A$ (eqn 3). The mean total number of larvae hatched in week *w*, μ_{H_w}, is estimated by:

$$\mu_{H_w} = \sum_{c \in w} (\lambda_{c-T_H,U} \tilde{A}_{c-T_H,U} + \lambda_{c-T_H,W} \tilde{A}_{c-T_H,W}) / n_w \qquad \text{(S6.9)}$$

where n_w is the number of days in week *w* on which eggs were hatched.

The observed mean number of larvae that hatch in week *w*, H_w, is assumed to follow a normal distribution $N(\mu_{H_w}, \sigma_H)$. The *Wolbachia* frequency in each cohort *c* follows a beta distribution, $\text{Beta}(n_{c,W}^L + 1, n_{c,U}^L + 1)$, where $n_{c,W}^L$ and $n_{c,U}^L$ are the numbers of newly-hatched infected and uninfected larvae observed in a sample from cohort *c*. We use these beta distributions to estimate the distribution of the *Wolbachia* frequency in the larvae hatched in week *w*, $\bar{f}_w^L$,

using numerical methods, and denote this distribution $P\left(\bar{f}_w^L\right)$. The likelihood is then given by:

$$p(\{n_{c,U}^L\},\{n_{c,W}^L\},\{H_c\} \mid \{L_i\},\{P_i\},\{\bar{f}_w^P\},\Omega) = \prod_{w=1}^{W} P(\bar{f}_w^L) N(H_w,\mu_{H_w},\sigma_H) \quad \text{(S6.10)}$$

where Ω is the vector of constant model parameters $\Omega = [a_W, a_U, b_W, b_U]$, W is the total number of weeks and

$$\bar{f}_w^L = \frac{\sum_{c \in w} \lambda_{c-T_H,W} \tilde{A}_{c-T_H,W}}{\sum_{c \in w} \lambda_{c-T_H,W} \tilde{A}_{c-T_H,W} + \lambda_{c-T_H,U} \tilde{A}_{c-T_H,U}} \quad \text{(S6.11)}$$

The posterior distribution is proportional to the product of the likelihood (eqn S6.10) and the prior $p(\Omega)$. We use uniform prior distributions: $U(0,\infty)$ for a_W and a_U ; $U(28,40)$ for b_W and b_U; $U(10,300)$ for σ_H.

We use a Metropolis-Hastings MCMC algorithm to sample from the posterior, with a fat-tailed student *t* distribution as the proposal distribution. For each parameter the *j*th proposal is given by:

$$a_W^{(j)} = a_W^{(j-1)} + c_1\varepsilon_1; \quad a_U^{(j)} = a_U^{(j-1)} + c_2\varepsilon_2$$
$$b_W^{(j)} = b_W^{(j-1)} + c_3\varepsilon_3; \quad b_U^{(j)} = b_U^{(j-1)} + c_4\varepsilon_4$$
$$\sigma_H^{(j)} = \sigma_H^{(j-1)} + c_5\varepsilon_5 \quad \text{(S6.12)}$$

where $\varepsilon_1,....,\varepsilon_5$ are random variables drawn from a student *t* distribution with 0.8 degrees of freedom. For Population A, c_2=0.4; c_4=0.05; c_6=25.0 and for Population B c_1=0.1; c_2=0.1; c_3=1.0; c_4=1.0 ; c_5=75.

S7. MCMC convergence results

(i) Estimating larval development time distributions

We ran the Metropolis-Hastings algorithm for 3 different chains each with different initial values for the 12 parameters. The initial value for each parameter was a random draw from its prior distribution. The Gelman-Rubin plots for all parameters showed that the three chains converged after 150000 updates for Population A (Additional file 11: Fig. S7.1) and after 250000 updates for Population B (Additional file 11: Fig. S7.1). These updates were treated as burn in and discarded from posterior analysis. For Population A the average acceptance rate for each parameter was between 0.16-0.57 with the exception of one parameter (ν_W) for which the average acceptance rate was 0.67. For Population B the average acceptance rate for each parameter was between 0.1-0.43 with the exception of one parameter (η_W) for which the average acceptance rate was 0.07.

(ii) Estimating per-capita female fecundity

We ran the Metropolis-Hastings algorithm for 3 different chains each with different initial values for the 3 parameters. The initial value for each parameter was a random draw from its prior distribution. The Gelman-Rubin plots for all parameters showed that the three chains converged after 50000 updates for both Population A (Additional file 12: Fig. S7.2) and Population B (Additional file

12: Fig. S7.2). These updates were treated as burn in and discarded from posterior analysis. The average acceptance rate for each parameter was between 0.2-0.44.

REFERENCES

Hancock, P.A., Linley-White, V., Callahan, A.G., Godfray, H.C.J., Hoffmann, A.A. & Ritchie, S.A. (2016) Density-dependent population dynamics in *Aedes aegypti* slow the spread of *w*Mel *Wolbachia*. *Journal of Applied Ecology,* **53,** 785-793.

Walker, T., Johnson, P.H., Moreira, L.A., Iturbe-Ormaetxe, I., Frentiu, F.D., McMeniman, C.J., Leong, Y.S., Dong, Y., Axford, J., Kriesner, P., Lloyd, A.L., Ritchie, S.A., O'Neill, S.L. & Hoffmann, A.A. (2011) The wMel *Wolbachia* strain blocks dengue and invades caged *Aedes aegypti* populations. *Nature,* **476,** 450-U101.
