# Supplementary material for: Predicting Wolbachia invasion dynamics in Aedes aegypti populations using models of density-dependent demographic traits
Source: BMC Biol. 2016 Nov 8;14:96. doi: 10.1186/s12915-016-0319-5 (PMC5100186; doi:10.1186/s12915-016-0319-5)
Supplement: Additional file 3: Figure S1.1. — Observed mosquito and wMel Wolbachia dynamics and the posterior fitted values. Red lines show the Markov Chain Monte Carlo iteration with the highest posterior probability and blue shaded areas show the 95 % credible interval. Results for Populations A and B are on the left and right of the dashed vertical line respectively. (A) Red circles show the observed wMel frequency in the pupae that eclosed in each week and vertical lines are the exact binomial 95 % confidence intervals. (B) Black circles show the observed weekly average pupal eclosion (PDF). (PDF 108 kb) [file 12915_2016_319_MOESM3_ESM.pdf]

Predicting Wolbachia invasion dynamics in Aedes aegypti populations using models of density-dependent demographic traits

Penelope A. Hancock, Vanessa L. White, Scott A. Ritchie, Ary A. Hoffmann, H. Charles J. Godfray

BMC Biology 2016

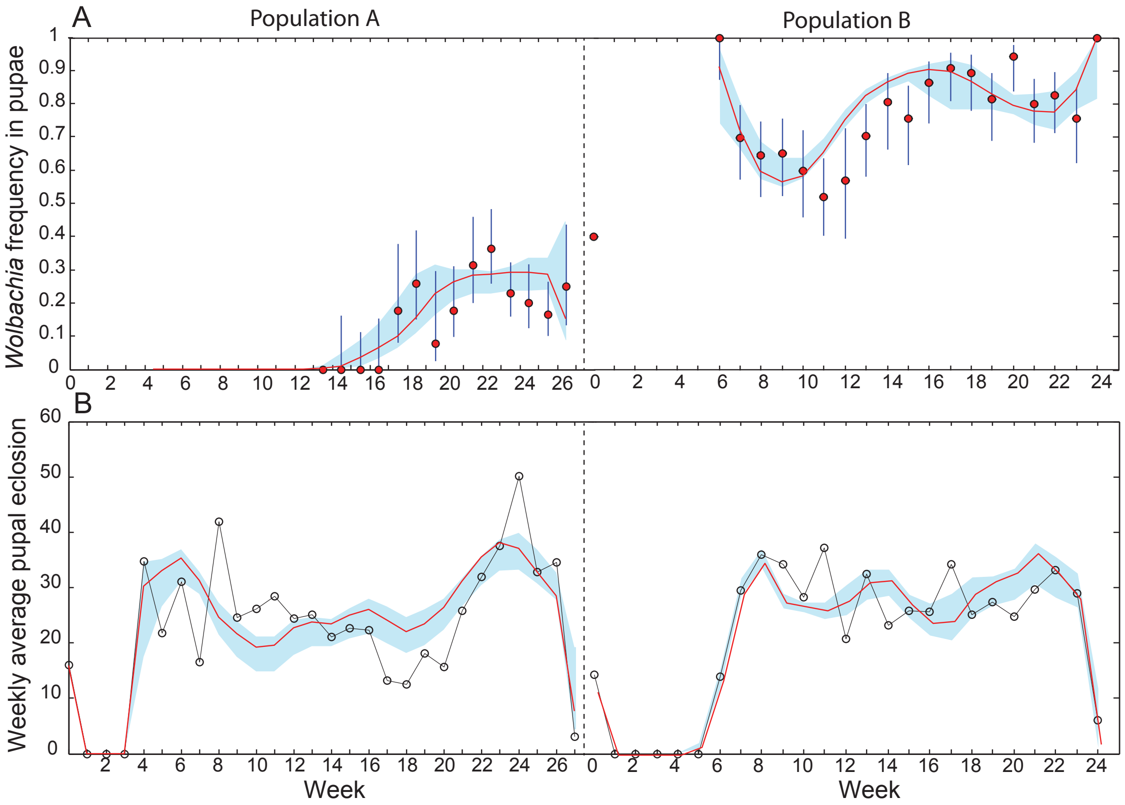

Additional file 3: Figure S1.1. Observed mosquito and *w*Mel *Wolbachia* dynamics and the posterior fitted values. Red lines show the MCMC iteration with the highest posterior probability and blue shaded areas show the 95% credible interval. Results for Population A and B are on the left and right of the dashed vertical line respectively. **(A)** Red circles show the observed *w*Mel frequency in the pupae that eclosed in each week and vertical lines are the exact binomial 95% confidence intervals. **(B)** Black circles show the observed weekly average pupal eclosion.
