# Supplementary material for: Predicting Wolbachia invasion dynamics in Aedes aegypti populations using models of density-dependent demographic traits
Source: BMC Biol. 2016 Nov 8;14:96. doi: 10.1186/s12915-016-0319-5 (PMC5100186; doi:10.1186/s12915-016-0319-5)
Supplement: Additional file 4: Figure S1.2. — The observed cumulative number of pupae (red lines) and hatched larvae (black lines) compared and the posterior fitted values. The predicted cumulative numbers of pupae (blue lines) and hatched larvae (green lines) are given by the maximum posterior probability iteration. Blue and green shaded areas show the 95 % credible interval for the cumulative numbers of pupae and hatched larvae respectively. Results for Populations A and B are shown on the left and right side of the vertical dotted line. (PDF 65 kb) [file 12915_2016_319_MOESM4_ESM.pdf]

Predicting Wolbachia invasion dynamics in Aedes aegypti populations using models of density-dependent demographic traits

Penelope A. Hancock, Vanessa L. White, Scott A. Ritchie, Ary A. Hoffmann, H. Charles J. Godfray

BMC Biology 2016

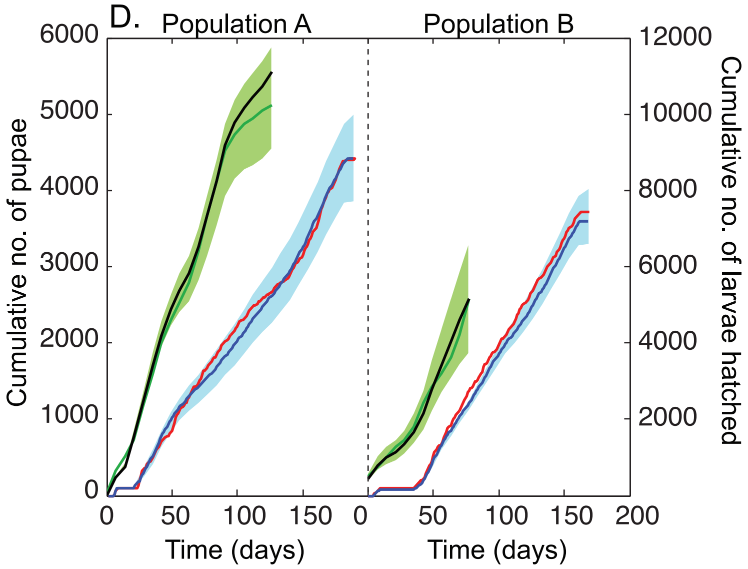

Additional file 4: Fig. S1.2. The observed cumulative number of pupae (red lines) and hatched larvae (black lines) compared and the posterior fitted values. The predicted cumulative numbers of pupae (blue lines) and hatched larvae (green lines) are given by the maximum posterior probability iteration. Blue and green shaded areas show the 95% credible interval for the cumulative numbers of pupae and hatched larvae respectively. Results for Populations A and B are shown on the left and right side of the vertical dotted line.
