# Supplementary material for: Predicting Wolbachia invasion dynamics in Aedes aegypti populations using models of density-dependent demographic traits
Source: BMC Biol. 2016 Nov 8;14:96. doi: 10.1186/s12915-016-0319-5 (PMC5100186; doi:10.1186/s12915-016-0319-5)
Supplement: Additional file 5: Figure S1.3. — Observed numbers of larvae hatched and the posterior fitted values. Red lines show the Markov Chain Monte Carlo iteration with the highest posterior probability and blue shaded areas show the 95 % credible interval. Results for Population A and B are on the left and right of the dashed vertical line, respectively. Black circles show the observed weekly average total number of larvae hatched. (PDF 62 kb) [file 12915_2016_319_MOESM5_ESM.pdf]

**Predicting Wolbachia invasion dynamics in Aedes aegypti populations using models of density-dependent demographic traits**

Penelope A. Hancock, Vanessa L. White, Scott A. Ritchie, Ary A. Hoffmann, H. Charles J. Godfray

*BMC Biology 2016*

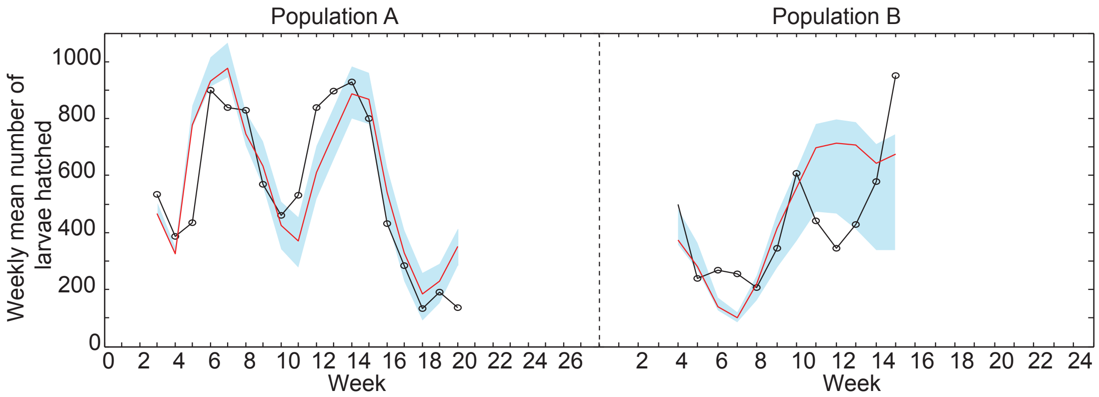


**Additional file 5: Figure S1.3.** Observed numbers of larvae hatched and the posterior fitted values. Red lines show the MCMC iteration with the highest posterior probability and blue shaded areas show the 95% credible interval. Results for Population A and B are on the left and right of the dashed vertical line respectively. Black circles show the observed weekly average total number of larvae hatched.
