# Supplementary material for: Predicting Wolbachia invasion dynamics in Aedes aegypti populations using models of density-dependent demographic traits
Source: BMC Biol. 2016 Nov 8;14:96. doi: 10.1186/s12915-016-0319-5 (PMC5100186; doi:10.1186/s12915-016-0319-5)
Supplement: Additional file 6: Figure S2.1. — Posterior distributions of the differences in means (μ c,U − μ c,W; blue lines) and standard deviations (σ c,U − σ c,W; red lines) of the development time distributions of infected and uninfected larvae hatched in each cohort. For Population A, results for cohorts 47, 48 and 52 are not shown because the observed infection frequency in these first instar larvae was close to 0. For Population B, results for cohorts 19, 22 and 26 are not shown because the observed infection frequency in these first instar larvae was close to 1. (PDF 238 kb) [file 12915_2016_319_MOESM6_ESM.pdf]

Predicting Wolbachia invasion dynamics in Aedes aegypti populations using models of density-dependent demographic traits

Penelope A. Hancock, Vanessa L. White, Scott A. Ritchie, Ary A. Hoffmann, H. Charles J. Godfray

BMC Biology 2016

Population A

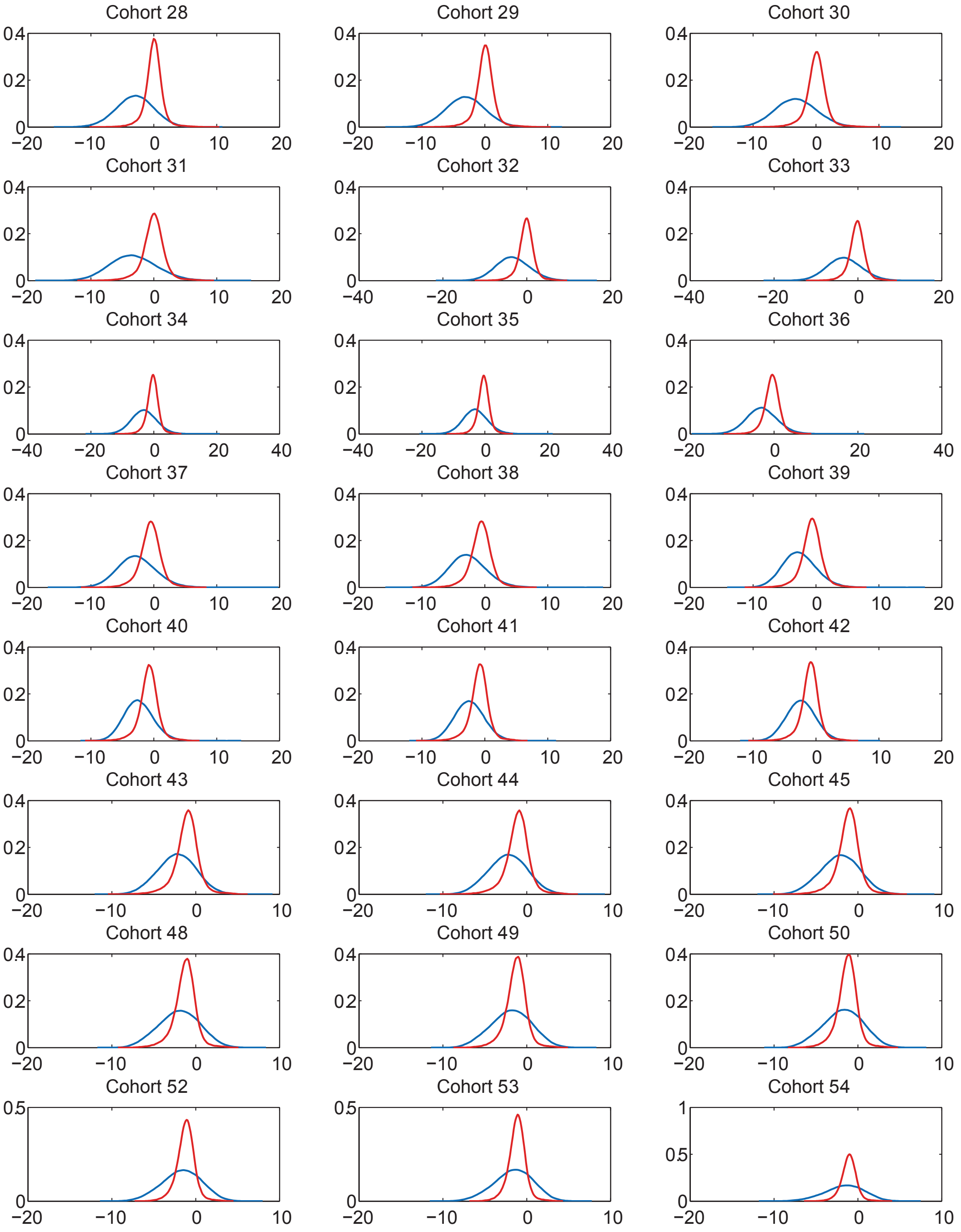

Population B

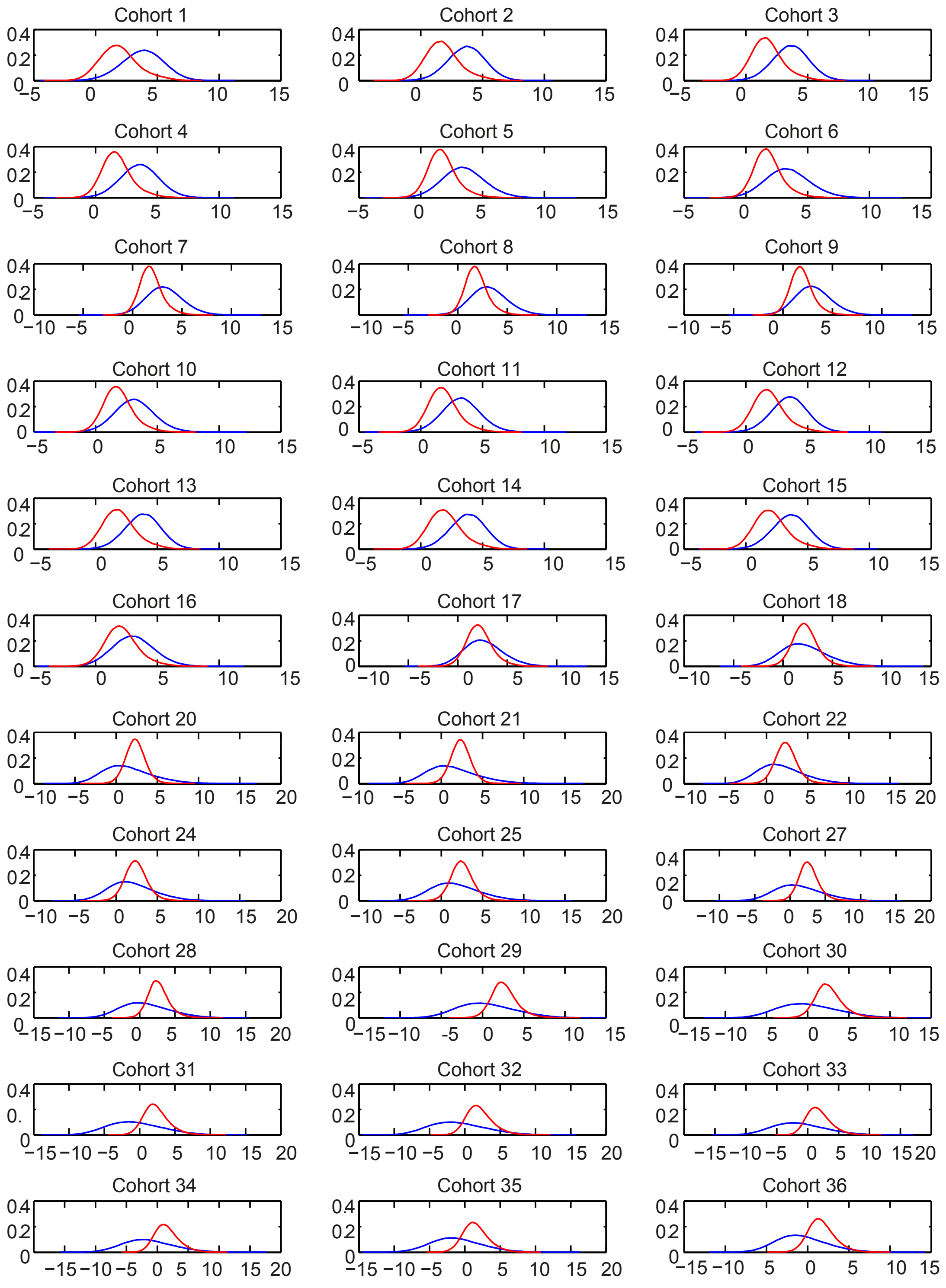

Additional file 6: Figure S2.1. Posterior distributions of the differences in means ($\mu_{c,U} - \mu_{c,W}$; blue lines) and standard deviations ($\sigma_{c,U} - \sigma_{c,W}$; red lines) of the development time distributions of infected and uninfected larvae hatched in each cohort. For Population A, results for cohorts 47, 48 and 52 are not shown because the observed infection frequency in these first instar larvae was close to 0. For Population B, results for cohorts 19, 22 and 26 are not shown because the observed infection frequency in these first instar larvae was close to 1.
