# Supplementary material for: Predicting Wolbachia invasion dynamics in Aedes aegypti populations using models of density-dependent demographic traits
Source: BMC Biol. 2016 Nov 8;14:96. doi: 10.1186/s12915-016-0319-5 (PMC5100186; doi:10.1186/s12915-016-0319-5)
Supplement: Additional file 7: Figure S3.1. — Observed wMel Wolbachia dynamics in the first instar larvae and the posterior fitted values, for Population B. The red lines show the Markov Chain Monte Carlo iteration with the highest posterior probability and blue shaded area shows the 95 % credible interval. Red circles show the observed wMel frequency in the first instar larvae that hatched in each week and vertical lines are the exact binomial 95 % confidence intervals. (PDF 61 kb) [file 12915_2016_319_MOESM7_ESM.pdf]

Predicting Wolbachia invasion dynamics in Aedes aegypti populations using models of density-dependent demographic traits

Penelope A. Hancock, Vanessa L. White, Scott A. Ritchie, Ary A. Hoffmann, H. Charles J. Godfray

BMC Biology 2016

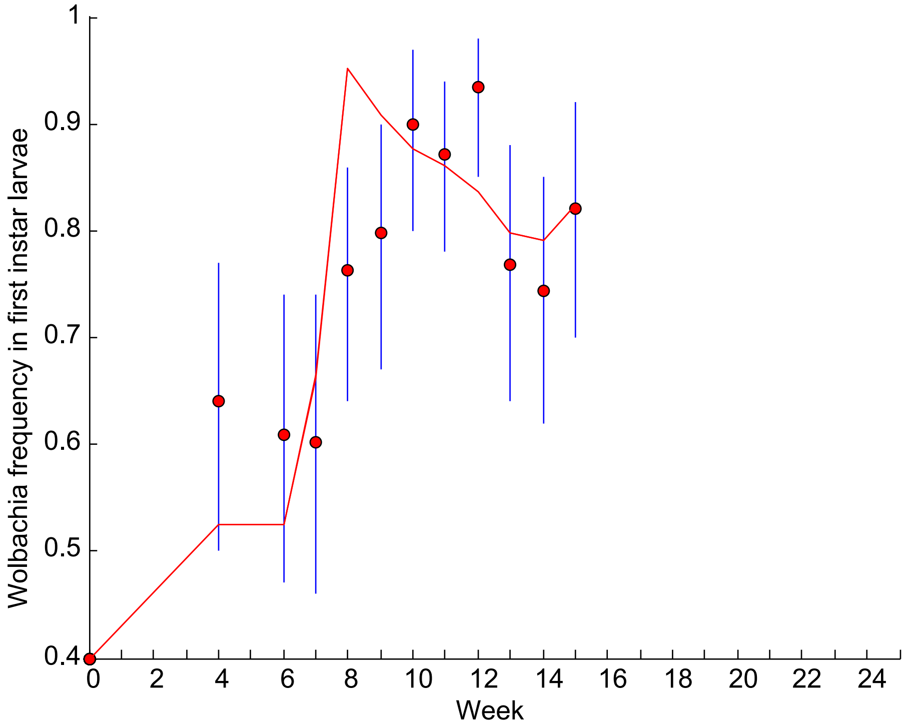

Additional file 7: Figure S3.1. Observed *w*Mel *Wolbachia* dynamics in the first instar larvae and the posterior fitted values, for Population B. The red lines show the MCMC iteration with the highest posterior probability and blue shaded area shows the 95% credible interval. Red circles show the observed *w*Mel frequency in the first instar larvae that hatched in each week and vertical lines are the exact binomial 95% confidence intervals.
