# Supplementary material for: Predicting Wolbachia invasion dynamics in Aedes aegypti populations using models of density-dependent demographic traits
Source: BMC Biol. 2016 Nov 8;14:96. doi: 10.1186/s12915-016-0319-5 (PMC5100186; doi:10.1186/s12915-016-0319-5)
Supplement: Additional file 8: Figure S3.2. — Posterior distributions of the difference in the predicted per-capita fecundity of Wolbachia-infected and uninfected adult females (λ i,U − λ i,W) for each cohort for Population B. (PDF 119 kb) [file 12915_2016_319_MOESM8_ESM.pdf]

Predicting Wolbachia invasion dynamics in Aedes aegypti populations using models of density-dependent demographic traits

Penelope A. Hancock, Vanessa L. White, Scott A. Ritchie, Ary A. Hoffmann, H. Charles J. Godfray

BMC Biology 2016

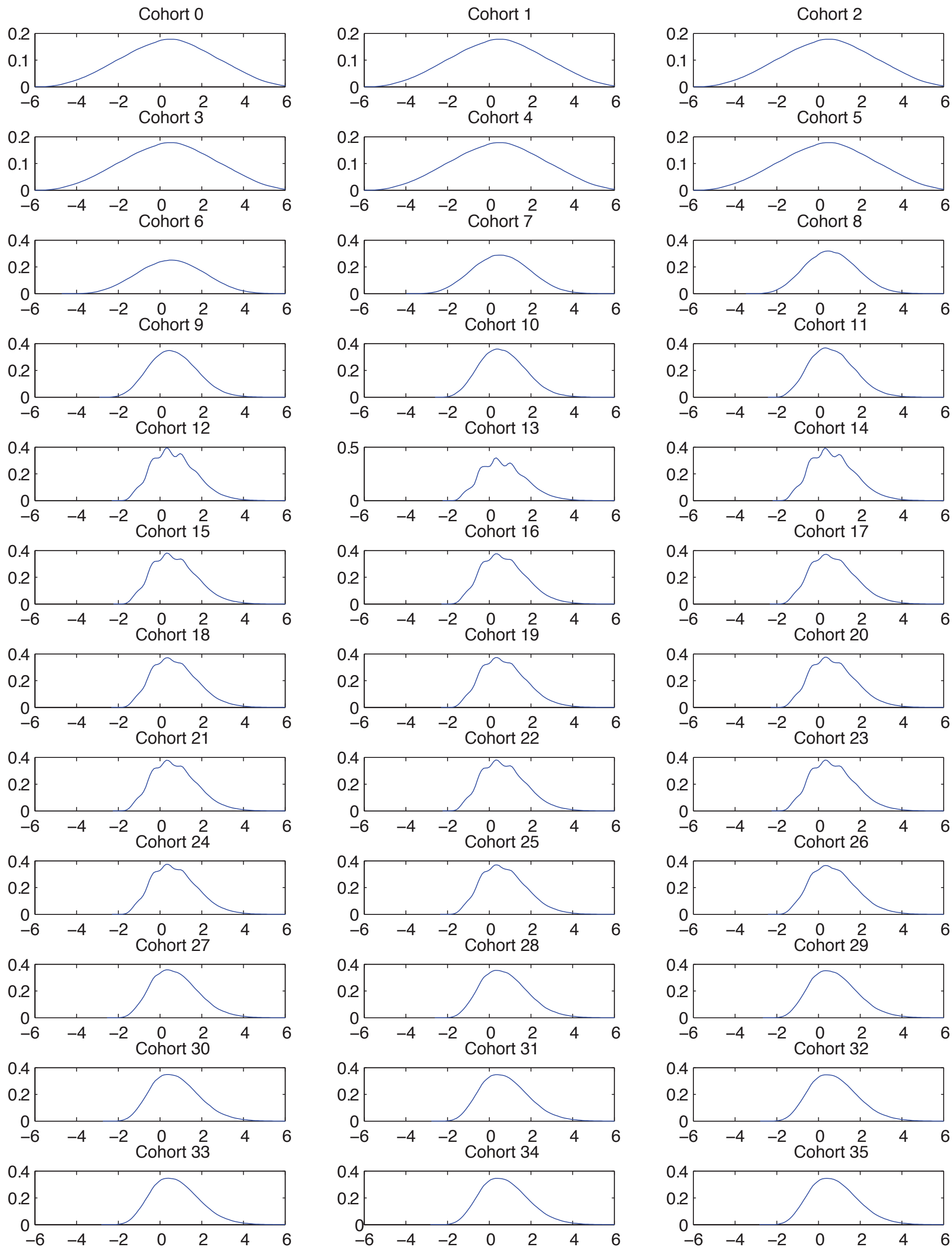

Additional file 8: Figure S3.2. Posterior distributions of the difference in the predicted per-capita fecundity of *Wolbachia*-infected and uninfected adult females ($\lambda_{i,U} - \lambda_{i,W}$) for each cohort for Population B.
