# Supplementary material for: Predicting Wolbachia invasion dynamics in Aedes aegypti populations using models of density-dependent demographic traits
Source: BMC Biol. 2016 Nov 8;14:96. doi: 10.1186/s12915-016-0319-5 (PMC5100186; doi:10.1186/s12915-016-0319-5)
Supplement: Additional file 9: Figure S5.1. — Total number of larvae counted in each week (black line and circles), divided into first and second instars (blue shading), third instars (yellow shading), and fourth instars (pink shading). A Population A; the red arrow indicates the day that introductions of wMel-infected pupae were initiated and the black arrow indicates the day that both wMel introductions and egg hatching were terminated (see [17]). B Population B; the black arrow indicates the day that egg hatching was terminated. (PDF 87 kb) [file 12915_2016_319_MOESM9_ESM.pdf]

Predicting Wolbachia invasion dynamics in Aedes aegypti populations using models of density-dependent demographic traits

Penelope A. Hancock, Vanessa L. White, Scott A. Ritchie, Ary A. Hoffmann, H. Charles J. Godfray

BMC Biology 2016

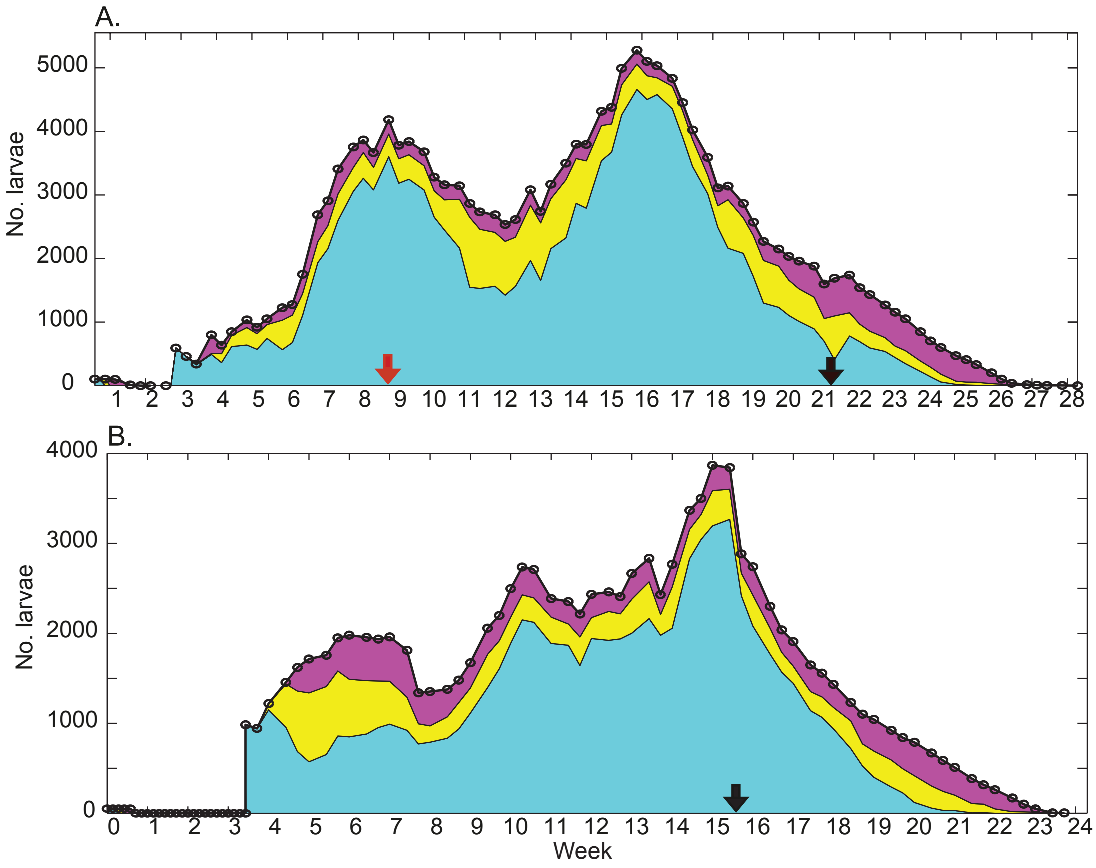

Additional file 9: Figure S5.1. Total number of larvae counted in each week (black line and circles), divided into first and second instars (blue shading), third instars (yellow shading) and fourth instars (pink shading). **A.** Population A; the red arrow indicates the day that introductions of *w*Mel-infected pupae were initiated and the black arrow indicates the day that both *w*Mel introductions and egg hatching were terminated (see (Hancock *et al.* 2016)). **B.** Population B; the black arrow indicates the day that egg hatching was terminated.
