# Supplementary material for: Predicting Wolbachia invasion dynamics in Aedes aegypti populations using models of density-dependent demographic traits
Source: BMC Biol. 2016 Nov 8;14:96. doi: 10.1186/s12915-016-0319-5 (PMC5100186; doi:10.1186/s12915-016-0319-5)
Supplement: Additional file 10: Figure S5.2. — The observed (black line and markers) and interpolated (red dashed line) larval survival over time. (PDF 57 kb) [file 12915_2016_319_MOESM10_ESM.pdf]

Predicting Wolbachia invasion dynamics in Aedes aegypti populations using models of density-dependent demographic traits

Penelope A. Hancock, Vanessa L. White, Scott A. Ritchie, Ary A. Hoffmann, H. Charles J. Godfray

BMC Biology 2016

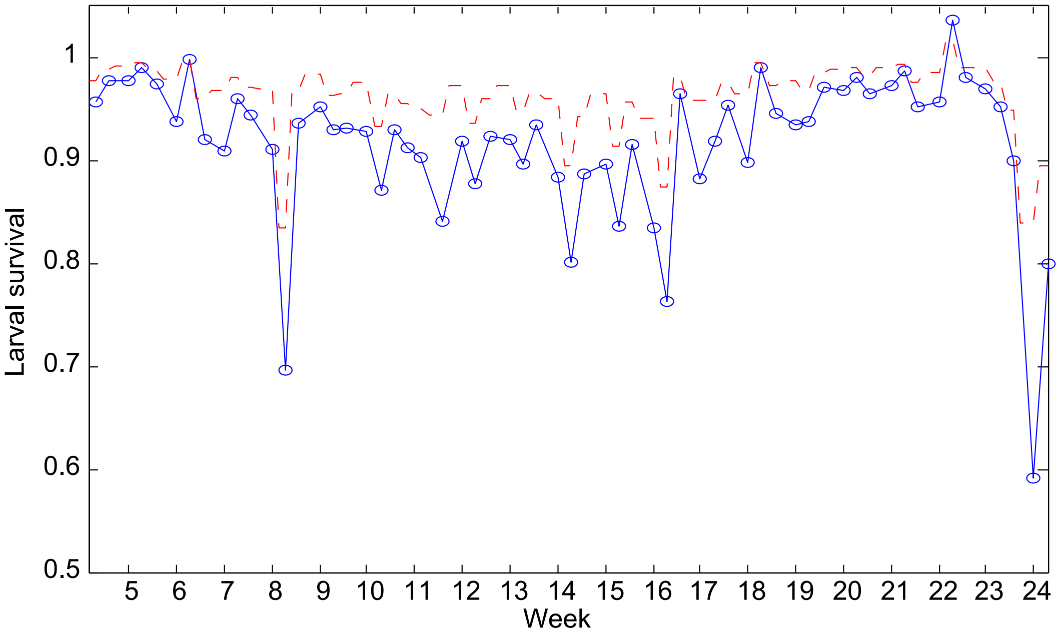

Additional file 10: Figure S5.2. The observed (black line and markers) and interpolated (red dashed line) larval survival over time.
