# Supplementary material for: Predicting Wolbachia invasion dynamics in Aedes aegypti populations using models of density-dependent demographic traits
Source: BMC Biol. 2016 Nov 8;14:96. doi: 10.1186/s12915-016-0319-5 (PMC5100186; doi:10.1186/s12915-016-0319-5)
Supplement: Additional file 11: Figure S7.1. — Gelman–Rubin plots of the posterior fitted values. Plots show the shrink factor for three chains each starting at different initial values: A, B. β U and β W; C, D. α U and α W; E, F. γ U and γ W; G, H. v U and v W; I, J. η U and η W; K, L. ψ U and ψ W. (PDF 199 kb) [file 12915_2016_319_MOESM11_ESM.pdf]

Predicting Wolbachia invasion dynamics in Aedes aegypti populations using models of density-dependent demographic traits

Penelope A. Hancock, Vanessa L. White, Scott A. Ritchie, Ary A. Hoffmann, H. Charles J. Godfray

BMC Biology 2016

Population A

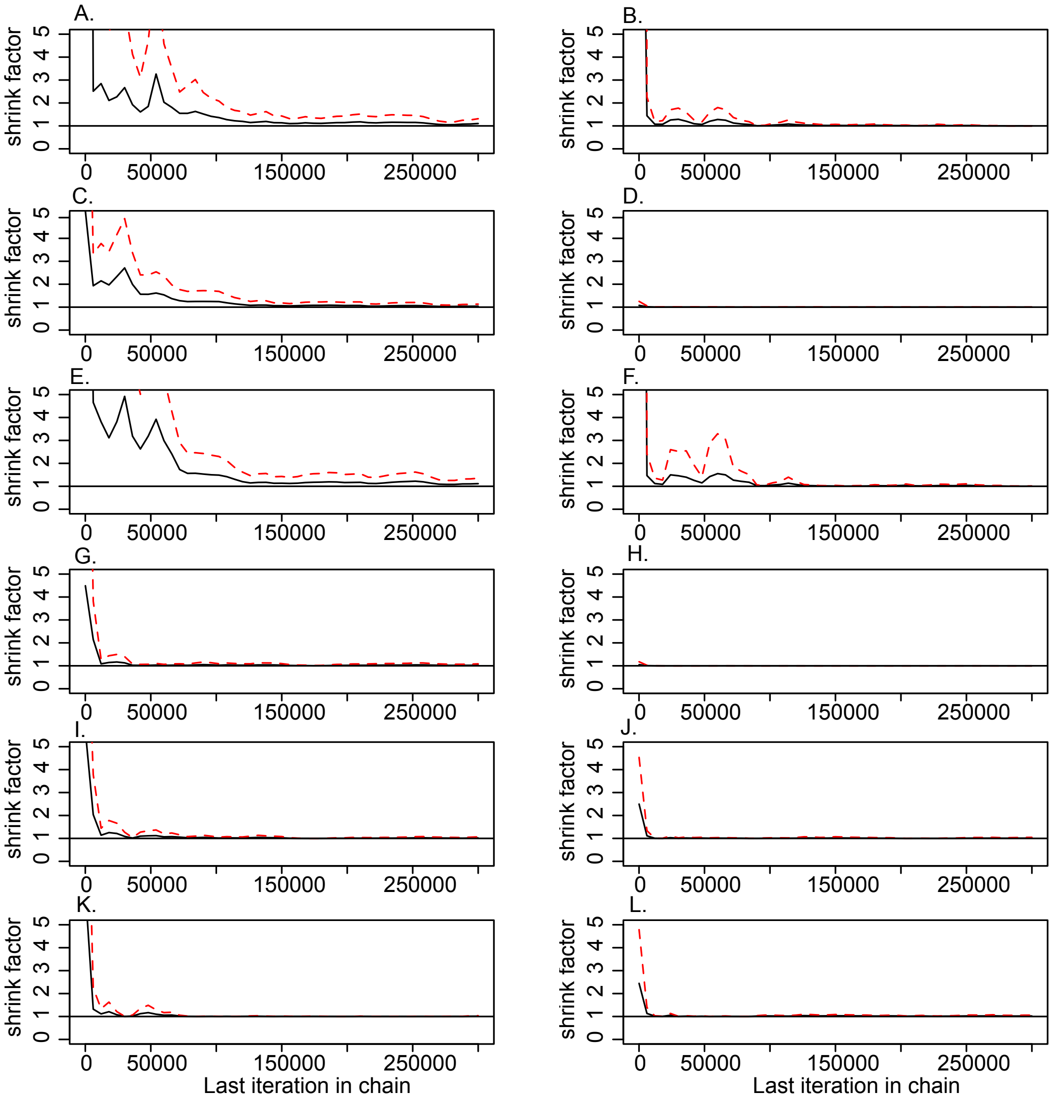

Population B

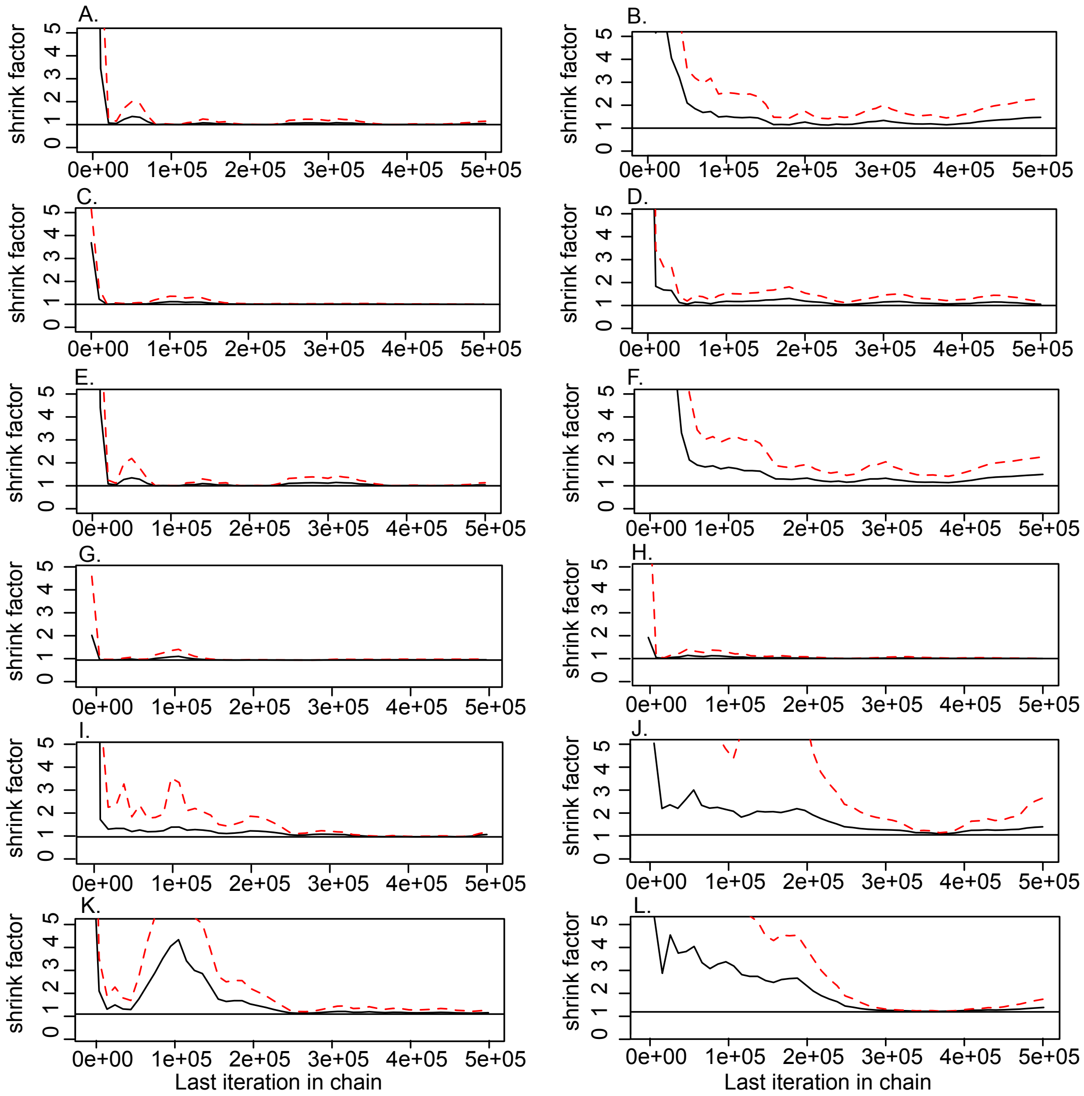

Additional file 11: Figure S7.1. Gelman-Rubin plots of the posterior fitted values. Plots show the shrink factor for three chains each starting at different initial values: A-B. β_U and β_W ; C-D. α_U and α_W ; E-F. γ_U and γ_W ; G-H. ν_U and ν_W ; I-J. η_U and η_W ; K-L. ψ_U and ψ_W .
