# Supplementary material for: Predicting Wolbachia invasion dynamics in Aedes aegypti populations using models of density-dependent demographic traits
Source: BMC Biol. 2016 Nov 8;14:96. doi: 10.1186/s12915-016-0319-5 (PMC5100186; doi:10.1186/s12915-016-0319-5)
Supplement: Additional file 12: Figure S7.2. — Gelman–Rubin plots showing the shrink factor for three chains each starting at different initial values: A. b; B. a. (PDF 71 kb) [file 12915_2016_319_MOESM12_ESM.pdf]

Predicting Wolbachia invasion dynamics in Aedes aegypti populations using models of density-dependent demographic traits

Penelope A. Hancock, Vanessa L. White, Scott A. Ritchie, Ary A. Hoffmann, H. Charles J. Godfray

BMC Biology 2016

Population A

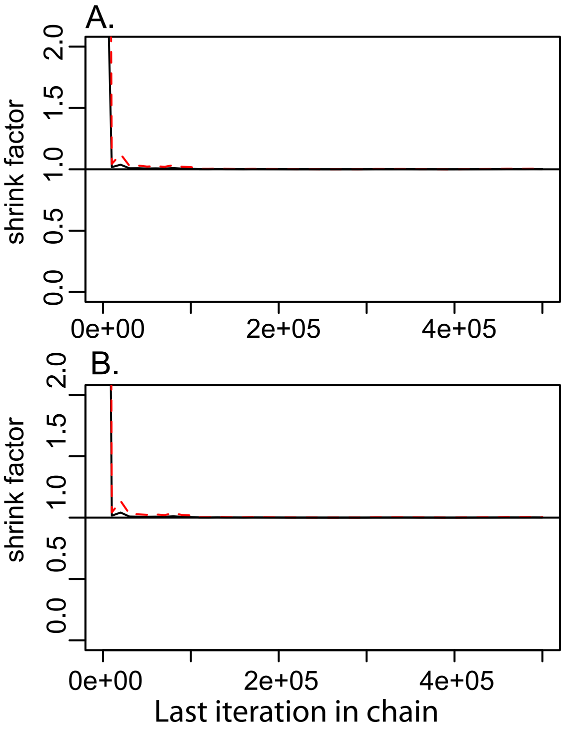

Population B

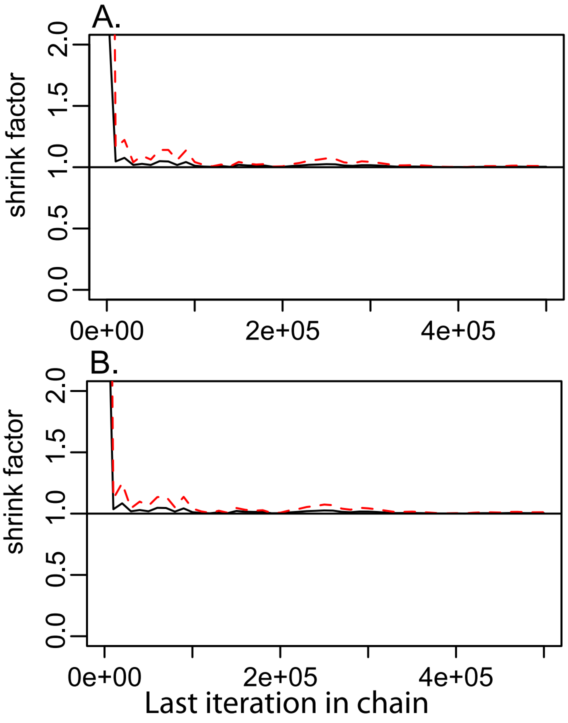

Additional file 12: Figure S7.2. Gelman-Rubin plots showing the shrink factor for three chains each starting at different initial values: A. *b*; B. *a*.
