# Supplementary material for: Predicting Wolbachia invasion dynamics in Aedes aegypti populations using models of density-dependent demographic traits
Source: BMC Biol. 2016 Nov 8;14:96. doi: 10.1186/s12915-016-0319-5 (PMC5100186; doi:10.1186/s12915-016-0319-5)
Supplement: Additional file 13: Figure S4.1. — Long term behaviour of the uninfected mosquito population in the absence of Wolbachia. A, B. The intensity of density-dependent competition is low (C L * = 0.018, μ I = 0.12). C, D. The intensity of density-dependent competition is high (C L * = 1.0, μ I = 0). A, C. Numbers of larvae (black line) and adults (red line). B, D. Per-capita adult female fecundity (red lines), larval development time mean (blue line) and standard deviation (black line). (PDF 107 kb) [file 12915_2016_319_MOESM13_ESM.pdf]

Predicting Wolbachia invasion dynamics in Aedes aegypti populations using models of density-dependent demographic traits

Penelope A. Hancock, Vanessa L. White, Scott A. Ritchie, Ary A. Hoffmann, H. Charles J. Godfray

BMC Biology 2016

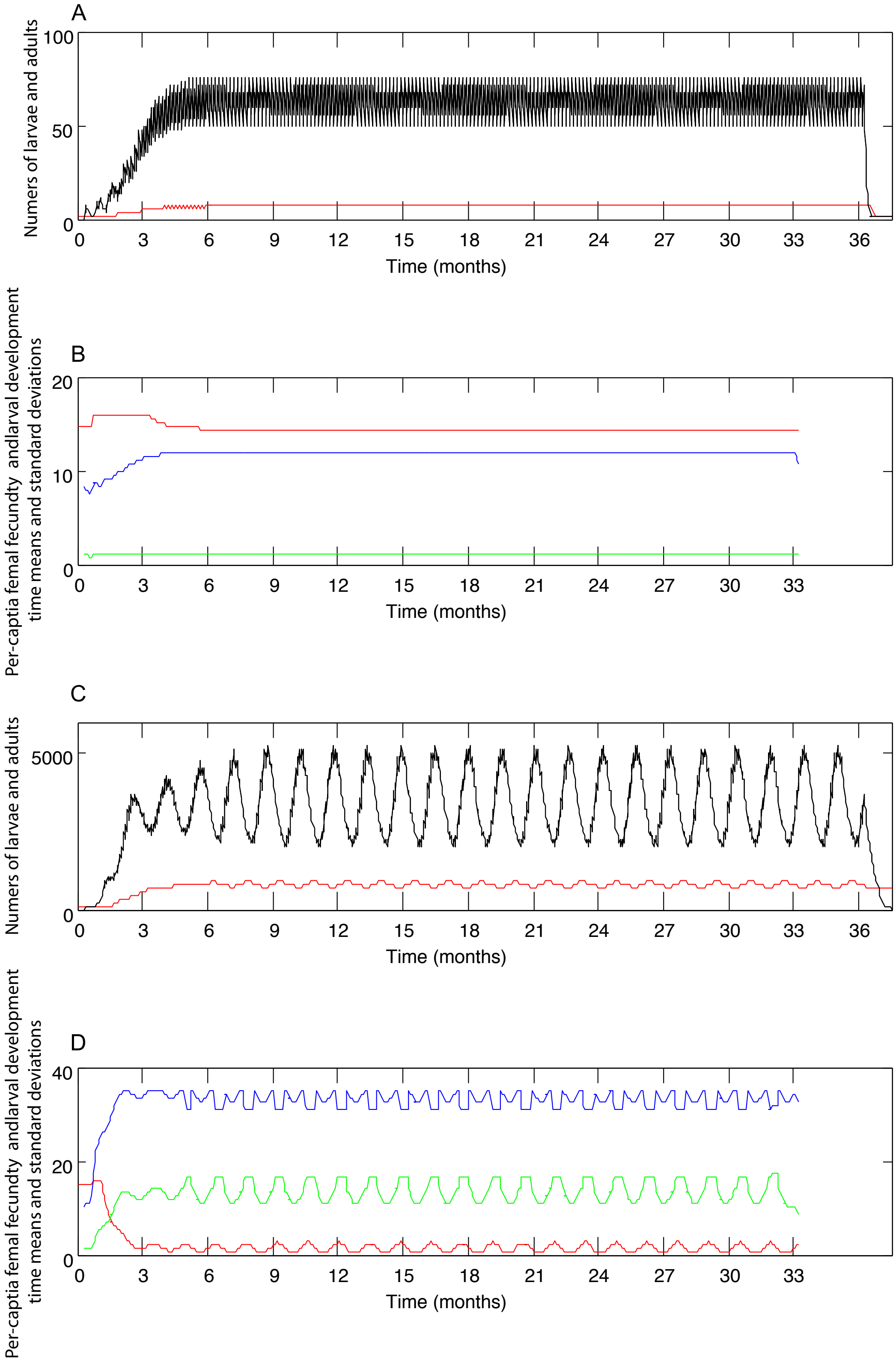

Additional file 13: Figure S4.1. Long term behaviour of the uninfected mosquito population in the absence of *Wolbachia*. A-B. The intensity of density-

dependent competition is low (C_L^*=0.018, μ_I=0.12); C-D. The intensity of density-dependent competition is high (C_L^*=1.0, μ_I=0). A,C. Numbers of larvae (black line) and adults (red line). B,D. Per-capita adult female fecundity (red lines), larval development time mean (blue line) and standard deviation (black line).
